# Supplementary material for: Effects of salt stress on the photosynthetic physiology and mineral ion absorption and distribution in white willow (Salix alba L.)
Source: PLoS One. 2021 Nov 18;16(11):e0260086. doi: 10.1371/journal.pone.0260086 (PMC8601552; doi:10.1371/journal.pone.0260086)
Supplement: Attachment — Submitted filename: PONE-D-21-23173_R1.pdf [file pone.0260086.s004.pdf]

PLOS ONE

Effects of salt stress on the photosynthetic physiology and mineral ion absorption and distribution in white willow(Salix alba L.)

--Manuscript Draft--

**Manuscript Number:**	PONE-D-21-23173R1
**Article Type:**	Research Article
**Full Title:**	Effects of salt stress on the photosynthetic physiology and mineral ion absorption and distribution in white willow(Salix alba L.)
**Short Title:**	Effects of salt stress on white willow(Salix alba L.)
**Corresponding Author:**	Bingxiang Liu<br>Hebei Agricultural University<br>Baoding, CHINA
**Keywords:**	Salix alba. L; NaCl stress; root growth; ion absorption and transport; photosynthetic system characteristics; chlorophyll fluorescence kinetics
**Abstract:**	[Objective] The purpose of this study was to explore the adaptive mechanism underlying the photosynthetic characteristics and the ion absorption and distribution of white willow (Salix alba L.) in a salt stress environment in cutting seedlings. The results lay a foundation for further understanding the distribution of sodium chloride and its effect on the photosynthetic system.[Method] A salt stress environment was simulated in a hydroponics system using different NaCl concentrations in one-year-old Salix alba L. cuttings as the test materials. The growth status of the cutting roots, ion absorption, transport and distribution in the roots and leaves, and the changes in the photosynthetic fluorescence parameters were studied after 20 days under hydroponics. [Results] The results show that root germination and elongation are promoted in the presence of 0.1% NaCl, but root growth is comprehensively inhibited under increasing salt stress. Under salt stress, Na^+ accumulates significantly in the roots and leaves, and the Na^+ content and the Na^+/K^+ and Na^+/Ca^{2+} root ratios are significantly greater than those in the leaves. When the NaCl concentration is ≤ 0.2%, Salix alba L. can maintain relatively stable K^+ and Ca^{2+} contents in its leaves by improving the selective absorption and accumulation of K^+ and Ca^{2+} and adjusting the transport capacity of mineral ions to aboveground parts while K^+ and Ca^{2+} levels are clearly decreased under high salt stress. With increasing salt concentrations, the net photosynthetic rate (P_n), transpiration rate (E) and stomatal conductance (g_s) of leaves decrease gradually overall, and the intercellular CO_2 concentration (C_i) first decreases and then increases. When the NaCl concentration is < 0.2%, the decrease in leaf P_n is primarily restricted by the stomata. When the NaCl concentration is > 0.2%, the decrease in the P_n is largely inhibited by non-stomatal factors. Due to the salt stress environment, the OJIP curve (Rapid chlorophyll fluorescence) of Salix alba L. turns into an OKJIP curve. When the NaCl concentration is > 0.1%, the fluorescence values of points I and P decrease significantly, which is accompanied by a clear inflection point (K). The quantum yield and energy distribution ratio of the PSII reaction center change significantly (φPo, Ψo and φEo show an overall downward trend while φDo is promoted). The performance index and driving force (PI_{ABS}, PI_{CSm} and DF_{CSm}) decrease significantly when the NaCl concentration is > 0.1%, indicating that salt stress causes a partial inactivation of the PSII reaction center, and the functions of the donor side and the recipient side are damaged. [Conclusion] The above results indicate that Salix alba L. can respond to salt stress by intercepting Na^+ in the roots, improving the selective absorption of K^+ and Ca^{2+} and the transport capacity to the above ground parts of the plant, and increasing φDo, thus showing an ability to self-regulate and adapt.
**Order of Authors:**	Xin Ran
	Xiao Wang
	Xiaokuan Gao
	Haiyong Liang
	Bingxiang Liu

Powered by Editorial Manager® and ProduXion Manager® from Aries Systems Corporation

	Xiaoxi Huang
**Response to Reviewers:**	Reply to all comments point-by-point (Manuscript Number: PONE-D-21-23173)<br><br>The authors' replies are in blue.<br><br><br>Editor's comments:<br><br>1. Please ensure that your manuscript meets PLOS ONE's style requirements, including those for file naming. The PLOS ONE style templates can be found at https://journals.plos.org/plosone/s/file?id=wjVg/PLOSOne_formatting_sample_main_body.pdf and https://journals.plos.org/plosone/s/file?id=ba62/PLOSOne_formatting_sample_title_authors_affiliations.pdf<br><br><br>[Reply] We have modified the manuscript according to the PLOS ONE's style requirements.<br><br>2. We note that you have stated that you will provide repository information for your data at acceptance. Should your manuscript be accepted for publication, we will hold it until you provide the relevant accession numbers or DOIs necessary to access your data. If you wish to make changes to your Data Availability statement, please describe these changes in your cover letter and we will update your Data Availability statement to reflect the information you provide.<br><br>[Reply] We will provide the relevant accession numbers or DOIs necessary to access our data.<br><br>3. PLOS requires an ORCID iD for the corresponding author in Editorial Manager on papers submitted after December 6th, 2016. Please ensure that you have an ORCID iD and that it is validated in Editorial Manager. To do this, go to 'Update my Information' (in the upper left-hand corner of the main menu), and click on the Fetch/Validate link next to the ORCID field. This will take you to the ORCID site and allow you to create a new iD or authenticate a pre-existing iD in Editorial Manager. Please see the following video for instructions on linking an ORCID iD to your Editorial Manager account: https://www.youtube.com/watch?v=_xcclfuvtxQ<br><br>[Reply] We create a new iD:https://orcid.org/0000-0002-1699-7648<br><br>COMMENTS FOR THE AUTHOR:<br>Reviewer 1#<br>- The study is carefully concepted and methodological approach is satisfactory explained. The manuscript deals with interesting and important changes in response to salt stress on the photosynthetic physiology and mineral ion absorption and distribution in white willow (Salix alba L.). Results and discussion portions are well written. The authors also draw an accurate picture from the results.<br><br>[Reply] Thank you very much for your valuable feedback. We have made corrections based on the suggestions. Please see the manuscript.<br><br>- However, few parameters are missing. The authors should review and cite some more relevant references, and follow suggestion given in detail below.<br><br>[Reply] We have reviewed and cited more relevant references. Please see line 472-495.<br><br>- Also needs a proper revision of English language. Many sentences are very confusing. English language and writing style needs to be improved sufficiently.<br><br>[Reply] We have modified the English language appropriately. Thank you very much for your valuable suggestions. We will continue our efforts to improve our English in the future.

- Do not repeat words of the title in the Keywords.

[Reply] We have rewrote Keywords. Please see line 41-43.

- Please for the first use Salix alba L., then please follow the only Salix alba in the manuscript.

[Reply] We have rewrote the name throughout the manuscript. Please look at the example from line 15+18.

- Add economic and other importance of plant in introduction to make it more valuable.

[Reply] We have added economic and other importance of plant in introduction. See Line 64-69 .

- It is better to NaCl concentration in mM instead of %.

[Reply] We've changed % to mM. Please see line 91.

- Line: 25- 27. Please site the reference for hydroponics.

[Reply] We've cited the reference for hydroponics. Please see Line 89 +92-95+441-447.

- Objective of the study needs to be refined.

[Reply] We have refined our research objectives. Please see Line 14-17.

- Line: 98. Which instrument (its name model, company) used for the determination of ions?
[Reply] We have added information about this instrument. We used the atomic absorption spectrometer of Analytikjena in Germany for atomic absorption determination.Please see line 116-117.

- In my opinion, chlorophyll content (a, b, and total) and carotenoids needs to be measured. It have direct link with salt stress and adaptability. NaCl and RWC have direct link when talking about stress and adaptability. So, I will suggest to study RWC (relative water content) under salt stress.
[Reply] This study mainly focuses on the effects of salt ions in plants on photosynthetic performance, without considering chlorophyII and water. Thank you for your valuable suggestions, which will be added and improved in our future studies.

- References need to be revised. Many articles on salinity response are published in high impact factor journals. So try to cite them, so everyone can access the references as well. Also, try to cite the latest articles.

[Reply] We have deleted some references. We have added some influential and recent articles. Please see line 472-495.

COMMENTS FOR THE AUTHOR:
Reviewer 2#
- However, this mechanism seems to be general in many plants, and also, as lack of data, the conclusions can not be rich and for further discussion.

[Reply] Thank you very much for your suggestions. We have revised the manuscript. Most of the researches on White Willow mainly focus on the medicinal value of substances such as salicin contained in the bark, or the value of studying the enrichment of heavy metals in white willow, which is mainly used to purify water resources and realize agricultural irrigation and fishery breeding. Habitat stress is mainly drought and flooding, but there are few literatures on salt stress, most of which focus on the responses of physiological indexes and photosynthetic indexes of plants to ion absorption and transport under salt stress. This is also one of the reasons for our

Powered by Editorial Manager® and ProduXion Manager® from Aries Systems Corporation

	research. We hope to use white willow as experimental material to observe the various effects of salt stress on plants from this perspective.<br><br><br>COMMENTS FOR THE AUTHOR:<br>Reviewer 3#<br>The manuscript titled “Effects of salt stress on the photosynthetic physiology and mineral ion absorption and distribution in white willow (Salix alba L.)” one of classic example of tree species response to salt stress. The manuscript is well organized and language easy to follow although there were few grammatical and Syntex errors besides SI units.<br><br>[Reply] Thank you very much for your suggestions. We have corrected grammatical and syntactic errors .Please see the manuscript.<br><br>-1. Adapting a hydroponic system to research salt stress may be the most recent best strategy, but there is no way of knowing how the experimental setting was done. Authors may include a photo of the same in order to make any relevant comments.<br><br>[Reply] We have added a picture to the manuscript. Please see the Figure 1.<br><br>-2.Similarly for rooting length, kindly provide some photo to understand plant response.<br><br>[Reply] We have added a photo. Please see the figure 2.<br><br>-3. Throughout the manuscript I can see that salt stress has a significant impact on above-ground biomass, such as leaf area index, shoot length, and so on, but the fact that it was not included in this study is a significant disadvantage. In the meantime, various physiological characteristics have been subjected leaves.<br><br>[Reply] This study mainly focuses on the effects of salt ions in plants on photosynthetic performance, without considering the aspects of leaves and stems. Thank you for your valuable suggestions, which will be added and improved in future research.<br><br>-4. When it comes to hydroponic systems, CK medium may be the best option, however, the reaction of Salix alba seedlings is substantially higher than 0.1% NaCl, but no justification from authors how or why although the salt concentration less than 0.1% NaCl.<br><br>[Reply] we have made an explanation, please see line 263-269.Thank you very much for your valuable suggestions.<br><br>-5. A statistical analysis was performed, however it was not up to scientific merit. For example, the manuscript frequently mentions significance of attributes, but there are no ANOVA tables (except LSD rank), at least as a supplemental to support. I request the authors to include F=xxx, df=xxx, and sig.=>0.001 wherever statistical significance was indicated.<br>[Reply] We added some these information throughout the manuscript. Please see the example form line 173-175+179+181.<br><br>6.Throughout the article, there are discussions about the trends of the morphological and physiological response of plant to the treatments, but the presented table does not provide viewpoints. Authors profusely mentioned the response trends widely without statistical analysis about trends which containing the scatter plot and best fit regression models (r2 and P). I advise authors to use graphs instead of tables, and I've included a sample presentation for ready reference.<br><br>[Reply] Thank you very much for your valuable suggestions. We have changed some tables into charts. Please see figure 7.
**Additional Information:**	
**Question**	**Response**

Powered by Editorial Manager® and ProduXion Manager® from Aries Systems Corporation

**Financial Disclosure**<br><br>Enter a financial disclosure statement that describes the sources of funding for the work included in this submission. Review the submission guidelines for detailed requirements. View published research articles from *PLOS ONE* for specific examples.<br><br>This statement is required for submission and **will appear in the published article** if the submission is accepted. Please make sure it is accurate.<br><br>**Unfunded studies**<br>Enter: *The author(s) received no specific funding for this work.*<br><br>**Funded studies**<br>Enter a statement with the following details:<br>• Initials of the authors who received each award<br>• Grant numbers awarded to each author<br>• The full name of each funder<br>• URL of each funder website<br>• Did the sponsors or funders play any role in the study design, data collection and analysis, decision to publish, or preparation of the manuscript?<br>• **NO** - Include this sentence at the end of your statement: *The funders had no role in study design, data collection and analysis, decision to publish, or preparation of the manuscript.*<br>• **YES** - Specify the role(s) played.<br><br>* typeset	This study was jointly supported by grants from the Natural Science foundation of Hebei Province(17226320D-4). The funders had no role in study design, data collection and analysis, decision to publish, or preparation of the manuscript.
**Competing Interests**<br><br>Use the instructions below to enter a competing interest statement for this submission. On behalf of all authors, disclose any competing interests that could be perceived to bias this work—acknowledging all financial support and any other relevant financial or non-financial competing interests.<br><br>This statement is required for submission	The authors have declared that no competing interests exist.

and **will appear in the published article** if the submission is accepted. Please make sure it is accurate and that any funding sources listed in your Funding Information later in the submission form are also declared in your Financial Disclosure statement.<br><br>View published research articles from *PLOS ONE* for specific examples.<br><br>**NO authors have competing interests**<br><br>Enter: *The authors have declared that no competing interests exist.*<br><br>**Authors with competing interests**<br><br>Enter competing interest details beginning with this statement:<br><br>*I have read the journal's policy and the authors of this manuscript have the following competing interests: [insert competing interests here]*<br><br>* typeset	
**Ethics Statement**<br><br>Enter an ethics statement for this submission. This statement is required if the study involved:<br><br>• Human participants<br>• Human specimens or tissue<br>• Vertebrate animals or cephalopods<br>• Vertebrate embryos or tissues<br>• Field research<br><br>Write "N/A" if the submission does not require an ethics statement.<br><br>General guidance is provided below. Consult the submission guidelines for detailed instructions. **Make sure that all information entered here is included in the Methods section of the manuscript.**	N/A

**Format for specific study types**<br><br>**Human Subject Research (involving human participants and/or tissue)**<br>• Give the name of the institutional review board or ethics committee that approved the study<br>• Include the approval number and/or a statement indicating approval of this research<br>• Indicate the form of consent obtained (written/oral) or the reason that consent was not obtained (e.g. the data were analyzed anonymously)<br><br>**Animal Research (involving vertebrate animals, embryos or tissues)**<br>• Provide the name of the Institutional Animal Care and Use Committee (IACUC) or other relevant ethics board that reviewed the study protocol, and indicate whether they approved this research or granted a formal waiver of ethical approval<br>• Include an approval number if one was obtained<br>• If the study involved *non-human primates*, add *additional details* about animal welfare and steps taken to ameliorate suffering<br>• If anesthesia, euthanasia, or any kind of animal sacrifice is part of the study, include briefly which substances and/or methods were applied<br><br>**Field Research**<br><br>Include the following details if this study involves the collection of plant, animal, or other materials from a natural setting:<br>• Field permit number<br>• Name of the institution or relevant body that granted permission	
**Data Availability**<br><br>Authors are required to make all data underlying the findings described fully available, without restriction, and from the time of publication. PLOS allows rare exceptions to address legal and ethical concerns. See the PLOS Data Policy and FAQ for detailed information.	Yes - all data are fully available without restriction

A Data Availability Statement describing where the data can be found is required at submission. Your answers to this question constitute the Data Availability Statement and **will be published in the article**, if accepted.<br><br>**Important:** Stating 'data available on request from the author' is not sufficient. If your data are only available upon request, select 'No' for the first question and explain your exceptional situation in the text box.<br><br>Do the authors confirm that all data underlying the findings described in their manuscript are fully available without restriction?	
**Describe where the data may be found in full sentences. If you are copying our sample text, replace any instances of XXX with the appropriate details.**<br><br>• If the data are **held or will be held in a public repository**, include URLs, accession numbers or DOIs. If this information will only be available after acceptance, indicate this by ticking the box below. For example: *All XXX files are available from the XXX database (accession number(s) XXX, XXX.*).<br>• If the data are all contained **within the manuscript and/or Supporting Information files**, enter the following: *All relevant data are within the manuscript and its Supporting Information files.*<br>• If neither of these applies but you are able to provide **details of access elsewhere**, with or without limitations, please do so. For example:<br><br>*Data cannot be shared publicly because of [XXX]. Data are available from the XXX Institutional Data Access / Ethics Committee (contact via XXX) for researchers who meet the criteria for access to confidential data.*<br><br>*The data underlying the results presented in the study are available from (include the name of the third party*	This is an open access article distributed under the terms of the Creative Commons Attribution License, which permits unrestricted use, distribution, and reproduction in any medium, provided the original author and source are credited.

and contact information or URL).<br>• This text is appropriate if the data are owned by a third party and authors do not have permission to share the data.<br><br>* typeset	
Additional data availability information:	Tick here if the URLs/accession numbers/DOIs will be available only after acceptance of the manuscript for publication so that we can ensure their inclusion before publication.

Powered by Editorial Manager® and ProduXion Manager® from Aries Systems Corporation

Manuscript
Click here to access/download;Manuscript;Manuscript.docx

Effects of salt stress on the photosynthetic physiology and mineral ion absorption and distribution in white willow (*Salix alba* L)

Xin Ran[1], Xiao Wang[1], Xiaokuan Gao[2], Haiyong Liang[1], Bingxiang Liu[1,3*], Xiaoxi Huang [1]

1. College of Forestry, Hebei Agricultural University , Baoding 071000, Hebei, China;

2. College of Life Science, Hengshui University, Hengshui 053000, Hebei, China;

3. Hebei Urban Forest Health Technology Innovation Center, Baoding 071000, Hebei, China

* Correspondence author.

E-mail address: proser211@126.com (Bingxiang Liu)

Abstract: [Objective] The purpose of this study was to explore the adaptive mechanism underlying the photosynthetic characteristics and the ion absorption and distribution of white willow (*Salix alba L*) in a salt stress environment in cutting seedlings. The results lay a foundation for further understanding the distribution of sodium chloride and its effect on the photosynthetic system. [Method] A salt stress environment was simulated in a hydroponics system using different NaCl concentrations in one-year-old *Salix alba*. cuttings as the test materials. The growth status of the cutting roots, ion absorption, transport and distribution in the roots and leaves, and the changes in the photosynthetic fluorescence parameters were studied after 20 days under hydroponics. [Results] The results show that root germination and elongation are promoted in the presence of 171mM/L NaCl, but root growth is comprehensively inhibited under increasing salt stress. Under salt stress, Na^+ accumulates significantly in the roots and leaves, and the Na^+ content and the Na^+/K^+ and Na^+/Ca^{2+} root ratios are significantly greater than those in the leaves. When the NaCl concentration is ≤ 342mM/L, Salix alba. can maintain relatively stable K^+ and Ca^{2+} contents in its leaves by improving the selective absorption and accumulation of K^+ and Ca^{2+} and adjusting the transport

capacity of mineral ions to aboveground parts, while K^+ and Ca^{2+} levels are clearly decreased under high salt stress. With increasing salt concentrations, the net photosynthetic rate (P_n), transpiration rate (E) and stomatal conductance (g_s) of leaves decrease gradually overall, and the intercellular CO_2 concentration (C_i) first decreases and then increases. When the NaCl concentration is < 342mM/L, the decrease in leaf P_n is primarily restricted by the stomata. When the NaCl concentration is > 342mM/L, the decrease in the P_n is largely inhibited by non-stomatal factors. Due to the salt stress environment, the OJIP curve (Rapid chlorophyll fluorescence) of *Salix alba*. turns into an OKJIP curve. When the NaCl concentration is > 171mM/L, the fluorescence values of points I and P decrease significantly, which is accompanied by a clear inflection point (K). The quantum yield and energy distribution ratio of the PS II reaction center change significantly (φPo, Ψo and φEo show an overall downward trend while φDo is promoted). The performance index and driving force (PI_{ABS}, PI_{CSm} and DF_{CSm}) decrease significantly when the NaCl concentration is > 171mM/L, indicating that salt stress causes a partial inactivation of the PSII reaction center, and the functions of the donor side and the recipient side are damaged. [Conclusion] The above results indicate that Salix alba. can respond to salt stress by intercepting Na^+ in the roots, improving the selective absorption of K^+ and Ca^{2+} and the transport capacity to the above ground parts of the plant, and increasing φDo, thus showing an ability to self-regulate and adapt.

Keywords: *Salix alba. L*; NaCl stress; root growth; ion absorption and transport; photosynthetic system characteristics; chlorophyll fluorescence kinetics

1 Introduction

With economic and social development, the problem of soil salinization has become increasingly prominent, resulting in approximately 1 billion hectares of saline-alkali land in the world[1]. The total area of China's saline-alkali land is believed to reach more than 100 million hectares[2]. Among the areas of concern, the coastal area, one of the primary types of saline-alkali land, has frequent water-salt interactions and secondary salinization because it is close to the sea[3]. Because the ecological environment of the salinization area is fragile and natural conditions are limited by many factors, it is highly significant to develop and use saline-alkali land scientifically and rationally while under pressure from a rapid population increase and a sharp decline in land resources; the goal is to advance towards the sustainable and healthy development of China's forestry and ecological environment [4].

Choosing and cultivating excellent salt-tolerant tree species through biotechnology is currently one of the most economical, effective, ecological and environmentally friendly biological measures to solve the soil salinization problem [5]. *Salix* is a deciduous tree or shrub belonging to the genus *Salix* in the Salicaceae. It has strong ecological adaptability and can grow well under saline-alkali, drought and barren soil conditions [6]. Previous studies on the salt tolerance of *Salix* plants were mostly focused on the physiological responses of seedlings to salt stress [7,8,9], but there are few studies on the adaptability of plants to salt stress specific to seedlings. High salt stress will cause plant water loss, ion imbalance and nutrient element deficiency through osmotic stress and ion poisoning [10], which will affect the normal growth and morphology of plants. A series of physiological growth changes in plants under salt stress are the comprehensive embodiment of their salt tolerance ability, among which the growth status of plant roots, the ion accumulation in different organs and the change in photosynthetic fluorescence parameters are important factors affecting the salt tolerance ability of plants [11,12,13]. These indicators can not only represent the extent of the effects of stress factors on plants, but they can also reflect the growth of plants under salt stress, the selective absorption and transport of ions, and the photosynthesis ability. Willow has the characteristics of antipyretic, analgesic, anti-inflammatory, anti-rheumatism, astringent, drought resistance[14] and anti-corrosion [15], among which the bark of White Willow contains salicin [16,17] with antibacterial, bactericidal, antioxidant, antipyretic, analgesic and other functions, and is a good natural food additive and food resource of health care products [18]. Its roots can also enrich harmful elements, reduce the impact of harmful elements on the surrounding soil [19,20], and play a role in purifying polluted water [21].*Salix alba.*has strong adaptability to adversity[22], so it has great potential for use and promotion in

the ecological management of coastal saline-alkali soil. Therefore, this experiment involved *Salix alba*. cuttings as the object and used hydroponics to simulate the seedling raising process of cuttings on coastal saline-alkali land to study the growth of their roots, the changing ion contents in the roots and leaves, and the changing photosynthetic fluorescence parameters under different salt concentrations. Exploring the characteristics of *Salix alba.*'s salt tolerance would provide a theoretical reference for research on its salt stress adaptability mechanism and its use in coastal saline-alkali areas.

2 Materials and Methods

2.1 Test materials and test design

The test materials were collected from the germplasm resource nursery of Golden Beach Forest Farm in Huai'an County, Hebei Province. The branches of the *Salix alba.* were basically the same in terms of growth, and those that were robust and free of diseases and insect pests were selected after the leaves fell in December. The middle two-thirds of the selected branches were cut into 20 cm-long cuttings. The uppermost bud was 0.5-1 cm from the top of the cuttings. The upper cut was a flat cut, and the lower cut was an oblique cut. The experiment was performed in the Artificial Climate Room of Hebei Agricultural University, Baoding City, Hebei Province on December 23, 2019. The temperature of the climate room was set to 28 ℃/25 ℃ (light/dark); the LED cold light source maintained the light intensity at 1000 $\mu mol \cdot m^{-2} \cdot s^{-1}$; the photoperiod was 14 h/10 h (light/dark); and the humidity was 60%.

The test material was placed in a 55 cm×38 cm×15 cm (length ×width ×height) plastic box for hydroponic culture(Fig 1). The experiment consisted of 5 treatments, and each treatment was repeated 3 times. We set the concentration of Nacl and the composition of culture medium by referring to existing studies[23,24,25]. A 1/2 dilution of Hoagland's complete nutrient solution was used as the base to prepare hydroponic solutions with NaCl concentrations of 171mmol/L、342mmol/L、513mmol/L、684mmol/L, and 1/2 Hoagland's complete nutrient solution (PH=7.2) was used as a control (CK). 1/2 Hoagland's complete nutrient solution includes: $Ca(NO_3)_2 \cdot 4H_2O$ 472.5mg/L、K_2SO_4 303.5mg/L、$NH_4H_2PO_4$ 57.5mg/L、$MgSO_4$ 246.5mg/L、$NaFeC_{10}H_{12}N_2O_8 \cdot 3H_2O$ 30mg/L、$FeSO_4$ 15mg/L、H_3BO_3 2.86mg/L、$Na_2B_4O_7 \cdot 10H_2O$ 4.5mg/L、$MnSO_4$ 2.13mg/L, $CuSO_4$ 0.05mg/L、$ZnSO_4$ 0.22mg/L、$H_8MoN_2O_4$ 0.02mg/L. There were 25 cuttings in each treatment, and they were soaked directly in the solution; the height of the solution was approximately more than half of the height of the cuttings. The nutrient solution was

changed every 5 days during the growth process. Before the nutrient solution was changed, the cuttings were removed and the roots were rinsed with water to wash away the last residual salt and prevent excessive salt accumulation. The contents of Na^+, K^+ and Ca^{2+} ions and the photosynthetic parameters and chlorophyll fluorescence kinetic curve parameters in the roots and leaves were determined following 20 days of treatment.

2.2 Measured items and methods

2.2.1 Measurement of root growth parameters

During the growth of *Salix alba.*, the number of root sprouting days and the rooting rate of all the cuttings were counted, and the rooting index was calculated according to the number of root sprouting days (rooting index = ∑Gt/Dt, where Dt, the day of the rooting test; Gt, the number of rooting branches on the day, and the rooting index is the number of rooting branches on the day/sum of days). After 20 days of salt stress treatment, 5 uniformly growing cuttings were selected to measure the average root number and average root length.

2.2.2 Determination of ion contents in the roots and leaves, calculation of the ion selective absorption and transport ratio

The measurement method for tracking the ion content was slightly modified relative to the method by Yang Sheng et al. [26] and Yu Bingjun et al. [27]. The sample was first baked at 105°C for 30 min and then dried at 70-80°C to a constant weight. After the sample was ground and passed through a sieve (the aperture was 0.425 mm), the fixed mass was weighed. Thirty mL of deionized water was added to the sample, which was then shaken well and placed in a boiling water bath for 2 h. After cooling, the sample was filtered and diluted to 50 mL. The Na^+, K^+ and Ca^{2+} contents were determined by atomic absorption method (Atomic absorption spectrometer: ZEEnit700-700P; analytikjena computer in Germany). The methods of Zheng Qingsong et al. [28] and Yang Xiaoying et al. [29] were used to calculate the selective absorption and transport coefficients of ions X (K^+ and Ca^{2+}) by the roots and leaves according to the following formula. Ion absorption coefficient $SA_{x, Na}$ =root ([X]/[Na^+])/medium ([X]/[Na^+]); ion transport coefficient ST x, $_{Na}$ = leaf ([X]/[Na^+])/Root ([X]/[Na^+]). In the formula, the K^+ content in the medium (culture broth) was 272 mg/L, and the Ca^{2+} content was 230 mg/L.

2.2.3 Determination of photosynthetic parameters in the leaves

Following 20 days of salt stress treatment, the photosynthetic gas exchange parameters of the *Salix alba*. leaves were measured. Five uniformly growing cuttings were selected for each treatment group in the test. After the cuttings were left under normal illumination in the climate room for 3 hours, we selected the 3rd to 5th leaves from the top to bottom with the same position, size, and light-receiving direction and with fully expanded functional leaves. Using a Li-6800 portable photosynthesis meter (LI-COR, USA), the P_n, E, g_s and C_i can be determined. The measurement conditions were as follows: the PAR was 1000 $\mu mol \cdot m^{-2} \cdot s^{-1}$, the CO_2 concentration in the fixed system was 400 $\mu mol \cdot mol^{-1}$, and the relative humidity was 60%.

2.2.4 Rapid determination of the chlorophyll fluorescence induction kinetic curve

After 20 days of salt stress treatment, 5 *Salix alba*. cuttings with average growth were selected from each treatment for measurement. Before the measurement, the leaves were dark-adapted for 15 minutes, and then the rapid chlorophyll fluorescence induction kinetic curve and related parameters were measured using a Pocket PEA plant efficiency analyzer (Pocket PEA, Hansatech, UK). The resulting O-K-J-I-P curve was used for rapid chlorophyll fluorescence induction curve data analysis (JIP-test) and calculation [30,31].

2.3 data processing

One-way ANOVA and the LSD method were used to test the significance of the differences (α =0. 05).

3 Results

3.1 Effects of salt stress on *Salix albicans* root growth

The test results show that although plants under salt stress can reach a 100% rooting rate between treatments, the average root number, average root length and rooting index are quite different among the treatments, and the overall trend is basically the same. The trend is that low-salt stress stimulates root germination and elongation, high-salt stress inhibits root growth, and the intensity of the inhibition is positively correlated with the salt concentration.

Figs 2 and 3 show that when the NaCl concentration was 171mM/L, the average root number and average root length were significantly increased compared with those of the control. This result may be a stimulating effect of low-salt stress on root growth and then appear again as the stress intensifies, with a gradual downward trend. When

the NaCl concentration was 513mM/L, the root number and length were significantly lower than those of their respective controls by 48.7% and 39.9%, and the root growth was significantly inhibited at that time. Compared with the control, the rooting index did not change significantly when the NaCl concentration was 171mM/L, but with the increase in stress, the number of days for root germination was delayed, and the rooting index decreased significantly. When the NaCl concentrations were 342mM/L, 513mM/L and 684mM/L, the rooting indexes were significantly lower than that of the control (9.6%, 18.1% and 27.7%).

3.2 Effects of salt stress on ion content, absorption and transport in the roots and leaves of *Salix alba*

The ion content measurements (Fig 4) showed that under different concentrations of NaCl, the Na^+ contents in the roots and leaves of *Salix alba*. were significantly higher than that in the control group, and the range of Na^+ change was positively correlated with the stress concentration. The comparison of Na^+ contents in the roots and leaves shows that the Na^+ content of the roots is much higher than that in the leaves. Under 684mM/L NaCl stress, the Na^+ content in the roots could reach twice that in the leaves. With increasing stress concentration, the K^+ content in the leaves first increased and then decreased, reaching a peak at a concentration of 171mM/L NaCl, which was a significant increase of 14.0% compared to the control group. However, after the NaCl concentration was greater than 342mM/L, the concentration was significantly lower than that of the control. As the stress concentration increased, the K^+ contents in the roots of each treatment group showed a gradual decrease, which were all significantly lower than that of the control. The Ca^{2+} content in the leaves of *Salix alba*. increased first and then decreased with increasing salt concentration. At 342mM/L NaCl, compared with the control group, the concentration significantly increased by 13.6% and then showed a significant downward trend. The Ca^{2+} content in the roots decreased continuously with increasing stress, and when the NaCl concentration was 684mM/L, the Ca^{2+} content dropped to 35.6% of the control.

Figs 5 and 6 show that both the Na^+/K^+ and Na^+/Ca^{2+} in the roots and leaves increased significantly with increasing NaCl stress concentration. This finding shows that as the stress intensifies, the relative absorption of Na^+ by *Salix alba.* increases greatly, but the absorption of K^+ and Ca^{2+} decreases. The Na^+/K^+ and Na^+/Ca^{2+} contents of all the treatments gradually decreased from root to leaf, and the rising Na^+/K^+ (F=1263.766, df=4，Sig.<0.001) and Na^+/Ca^{2+} (F=10485.256, df=4，Sig.<0.001) in the roots were significantly higher than those in leaves (F=1235.223,

df=4，Sig.<0.001; F=2335.783, df=4，Sig.<0.001), suggesting that *Salix* willow could reduce the salt stress damage to young tissues by regulating ion transport.

As shown in Fig 7, with increasing NaCl stress, the $SA_{k,\ Na}$, $ST_{k,\ Na}$, $SA_{Ca,\ Na,}$ and $ST_{Ca,\ Na}$ all showed a trend of first increasing and then decreasing. When the NaCl concentration was less than or equal to 342mM/L, the selective absorption capacity of the roots for K^+ (F=998.922, df=4，Sig.<0.001)and Ca2+ (F=1018.689, df=4，Sig.<0.001) and the selective transport capacity of the leaves for K^+ (F=168.047, df=4，Sig.<0.001) and Ca^{2+} (F=29.925, df=4，Sig.<0.001) were enhanced and reached a significant level. The selective absorption capacity of roots for K^+ is greater than that of Ca^{2+}, but the selective transport capacity of the leaves to Ca^{2+} is greater than that of K^+. These results indicated that *Salix* willow could adjust the upward transport capacity of K^+ and Ca^{2+} via the selective absorption and accumulation of mineral ions to compensate for the change in concentration under salt stress, to prevent the impacts of nutrient deficiency and ion toxicity on the shoot growth.

3.3 Effects of salt stress on photosynthetic parameters in *Salix alba.*Leaves

Figs 8 and 9 show that the photosynthetic parameters of *Salix alba*. leaves were affected to different degrees under different salt concentrations. When the NaCl concentration was 171mM/L, the P_n of the leaves increased, but there was no significant difference from the control. Later, as the salt stress intensified, the photosynthetic carbon assimilation ability of *Salix alba*. leaves was significantly inhibited (F=95.66, df=4，Sig.<0.001); when the NaCl concentration was greater than 171mM/L, both the E (F=100.091, df=4，Sig.<0.001)and g_s (F=69.346, df=4，Sig.<0.001) were significantly lower than the control and became stronger; but at a low salt concentration (171mM/L NaCl), there is no significant difference from the control. With the increased salt concentration, the leaf C_i showed a trend of first decreasing and then increasing, reaching the lowest when the salt concentration was 342mM/L, which was significantly lower than the control by 10.4%, and then it gradually increased. The C_i of leaves (F=20.50, df=4，Sig.<0.001) under 513mM/L and 684mM/L NaCl treatments were not significantly different from that of the control, but they were significantly higher than the lowest value by 12.4% and 14.6%, respectively.

3.4 Effect of salt stress on the rapid chlorophyll fluorescence induction kinetic curve (OJIP) of *Salix alba*.Leaves

The OJIP curve can provide a great deal of photochemical information about PSⅡ and accurately reflect the state of the plant photosynthetic apparatus and the electron redox state of the PSⅡ donor side, acceptor side and PSⅡ reaction center in the photoreaction [32], thus representing the effects of external stress on the plant photosynthesis ability and even the degree of damage to the photosynthetic organs. Fig 10 shows that with of the increasing NaCl concentration, the OJIP curve of *Salix alba*. leaves changes to different degrees. Compared with the control group, under the 171mM/L NaCl treatment, the fluorescence value of JIP does not change significantly; when the NaCl concentration reaches 342mM/L and higher, the fluorescence values of I and P drop significantly and there is an obvious inflection point K (approximately 300 μs), and the OJIP curve changes to the O-K-J-I-P curve. The K-phase fluorescence value under high salt treatment is higher than that under low salt treatment, and the maximum fluorescence can be reached faster, which indicates that the higher the salt treatment concentration is, the greater the damage to the leaves of *Salix alba*.

3.5 Effects of salt stress on quantum yield and energy distribution ratio

Fig 11 shows that under different salt stresses, the energy absorbed, transformed, used for electron transfer, and dissipated by thermal radiation in the leaves of *Salix alba*. changes. Compared with the control group, with the increasing NaCl concentration, the maximum photochemical efficiency (φPo) of *Salix alba*. leaves after dark adaptation gradually decreased. Under the 342mM/L NaCl treatment, the φPo was significantly lower than that of the control. At that time, salt stress triggered photoinhibition, and the photosynthetic capacity of the leaves was reduced.

The excitons captured by the reaction center transfer electrons to the electron transport chain, and the ratio of excitons that exceed Q_A's other electron acceptors to promote Q_A reduction excitons (Ψo) and the light energy absorbed by the reaction center are used for electron transfer. The quantum yields (φEo) all increased first and then

decreased with the increasing salt stress. At 171mM/L NaCl, although the Ψo and φEo increased, they were not significantly different from the control. Later, as the stress intensified, both the Ψo and φEo were significantly lower than those of the control. When the NaCl concentration was 342mM/L, the Ψo and φEo were significantly lower than the 11.1% and 11.9% of the control group, respectively. Compared with the control group, salt stress increased the quantum ratio (φDo) of *Salix alba*. leaves for heat dissipation. When the NaCl concentration was 513mM/L, φDo was significantly higher than that of the control.

3.6 Influence of salt stress on the performance index and driving force

The performance index and driving force can accurately reflect the changes in the state of the photosynthetic apparatus of plants under stress. PI_{ABS} refers to the performance index based on the absorption of light energy, PI_{CSm} refers to the performance index based on the unit area, and DF_{CSm} refers to the driving force based on the unit area of the material. Figs 12 and 13 show that as the NaCl stress concentration increases, PI_{ABS}, PI_{CSm} and DF_{CSm} all show a gradual decline. PI_{ABS} showed no significant difference from the control when the NaCl concentration was 171mM/L, and then with the increased salt concentrations, the difference became more significant (F=61.074, df=4， Sig.<0.001) , indicating that the *Salix alba.* leaves experienced photoinhibition, the PSⅡ was damaged, and the measurement at the 684mM/L NaCl concentration was significantly lower than that of the control, by 60.2%. When the NaCl concentrations were 342mM/L, 513mM/L and 684mM/L, the PI_{CSm} values were significantly lower (F=202.821, df=4， Sig.<0.001) than that of the control by 20.1%, 43.9% and 66.4%; when the NaCl concentrations were 513mM/L and 684mM/L, the DF_{CSm} values were significantly lower (F=40.755, df=4， Sig.<0.001) than the control by 6.3% and 11.2%. Salt stress seriously affects the absorption of light energy by plants and leads to a decline in the basic driving force.

4 Conclusions and discussion

4.1 Influence of salt stress on the root growth status of *Salix alba*

As the primary organ responsible for plant material exchange, the root system and its growth status are closely related to the growth and development of the aboveground plant parts, whether the root system can function normally, and the plant's water and nutrient utilization efficiency [33]. Under salt stress, the root system is the first to feel the adversity stress signal, and it is also the most directly affected part [34]. Its ring-stripe inhibition is primarily manifested in the low levels of the root length, surface area and other parameters, and the root system grows slowly. A high-salt environment will cause plants to experience osmotic stress and ion toxicity, which will lead to changes in membrane permeability, which will in turn affect the absorption of water and nutrient elements by the roots, causing the plants to lose a large amount of water; the ions near the roots will be unbalanced, the physiological functions of the roots will eventually be lowered, and even the structure will be destroyed. Some of the aboveground leaves wilt, and photosynthetic production cannot be performed normally, which causes plant growth and metabolic disorders until the loss of physiological functions.

The change in root growth and the time of the root sprouting period can directly reflect the degree of damage to plants by salt stress and represent the strength of plant salt tolerance [35]. This study showed that the 171mM/L NaCl concentration significantly promoted the increase in the average number of roots and the elongation of the average root length of *Salix alba*. cuttings, and it can promote the rooting of the root system in advance, to a certain extent, which is consistent with Wang Shufeng et al. [36] and Ci Dun. The research results of Wei et al. [35] were basically the same. This growth response may be due to the decrease in water potential outside the roots under salt stress, which stimulates the growth of the roots instead of moderate osmotic stress to ensure the normal absorption of water and nutrients to meet the physiological and metabolic needs of the aboveground parts.

Some plants do have the phenomenon that low salt promotes the increase of some indicators, such as: promoting the germination of sorghum seeds[37], the roots of the seedlings of wolfberry[38] and rice[39], and the growth indicators of corn[40,41]. Both ChorophyⅡ in chrysanthemum [42] and proline content of cherry seedlings [43] are increased, while the net photosynthetic rate of wild chrysanthemum [44] and hazel trees increased [45]. The reason for the low salt concentration may be that the salt stress has a dual effect of stimulus and inhibition on plants. The strong and weak relationship between stimulus and inhibition triggers changes in various plant indicators, resulting in the same low salt. It can promote growth, and it will be inhibited after high salt.This finding shows that *Salix alba*. has some ability

to adjust and adapt to salt stress, and this adaptability is of great significance to the survival and continuation of the plant itself under adversity. However, as the salt stress intensifies, the ability of plants to coordinate their own growth is destroyed, root germination and elongation are significantly inhibited and become more intense, the root functions are destroyed, and the plants cannot maintain their normal growth and development.

4.2 Effects of salt stress on ion content, absorption and transport in *Salix alba*

Ions play an important role in the normal growth of plants, but salt stress can destroy the dynamic balance of ions in plants [46], hinder the absorption of nutrients, and cause plant metabolism disorders. The change in the distribution of ions reflects the degree of damage to plant cells by the external adverse environment. Additionally, plants can maintain balanced nutrition by improving the absorption and transport of ions, which also represents the level of plant resistance to stress. When measuring the ion contents of plant roots and leaves, it is helpful to reveal the salt tolerance or salt damage mechanism of plants.

In this study, when the salt concentration was low, the growth of *Salix alba*. was basically normal, the symptoms of salt damage were not significant, and the damage was obvious under severe stress. Na^+ accumulates significantly in the roots and leaves of *Salix alba*. under salt stress, but the Na^+ content in different organs is significantly different, and it is primarily concentrated in the roots. This result shows that the willow root system has a compensation mechanism that can reduce the transportation of salt to aboveground parts by enriching Na^+ in the root, thereby effectively reducing or delaying the occurrence of salt damage in the aboveground parts. This conclusion is consistent with the study by Hao Han et al. [47]. When the salt stress is too high, this balance is broken, and growth is blocked.

As an important inorganic solute, K^+ is essential for reducing the cell osmotic potential and maintaining the water balance. Generally, plants have an antagonistic effect on the absorption of Na^+ and K^+ [48], and the competition between the two usually leads to a decrease in the K^+ content. The loss of K^+ will cause changes in the physical structure of the stomata, frustrating photosynthesis [49]. In addition, K^+ participates in the metabolism of various enzymes in plants [50]. As salt stress increases, an excessive loss of K^+ will lead to K^+ dependent enzymes in *Salix alba*. The enzyme activity decreases, which affects the metabolic reactions in which it participates. Therefore, if

plants are to grow in a salty environment, the selective absorption of K^+ by the root system and the transportation of K^+ to the ground are particularly important. This study showed that the K^+ content in the roots of *Salix alba*. significantly decreased with increasing stress, but the K^+ in the leaves could be maintained at a high level at a 342mM/L NaCl concentration and below and even increased significantly when the NaCl concentration was 171mM/L, according to Zhou Qi et al. [51] A study on *Carpinus chinensis* also confirmed this result. At this time, the value and increase of Na^+/K^+ in the roots of the *Salix alba*. were greater than that of the leaves, and the $SA_{k,\ Na}$ and $ST_{k,\ Na}$ all increased significantly. Studies have shown that under salt stress, the Na^+/K^+ value can represent the degree of salt damage to the plant, and the lower Na^+/K^+ value of the leaves can help the plant better maintain its growth and photosynthetic function [52], and the $SA_{k,\ Na}$ and $ST_{k,\ Na}$ indicates that the plants can better tolerate salt stress [53]. This result shows that at that time, *Salix alba*. could maintain a relatively stable leaf K^+ content and the normal progress of photosynthesis by restricting the transportation of Na^+ from the root to the leaves, increasing the selective absorption of K^+ through the plant roots and the ability to transport K^+ to the ground. The accumulation of Na^+ causes damage to plants, which may be an important mechanism by which *Salix alba*. copes with salt stress. Later, with the increase in salt stress, the K^+ in the roots and leaves clearly flowed out. A high concentration of Na^+ will replace the Ca^{2+} bound to the membrane system, which will damage the integrity of the membrane structure and membrane function, thereby destroying the ion balance in the plant body and causing a large amount of organic solute extravasation [54]. The establishment of Ca^{2+} homeostasis in the cytoplasm is a key condition for salt adaptation [55]. This experiment showed that as the salt stress intensified, the Ca^{2+} content in the *Salix alba*. roots continued to decrease, but it could accumulate in the leaves when the NaCl concentration was ≤342mM/L. The results of Jia Yin et al. [56] were similar; the Na^+/Ca^{2+} value of white *Salix* roots was higher than that of the leaves, and the $Sa_{Ca,\ Na}$ and $ST_{ca,\ Na}$ were all significantly increased. This result may be due to the large influx of Na^+ into the root system under salt stress, activating Ca^{2+} signal transduction, triggering the sodium elimination system to reduce the damage of Na^+,

and enhancing the selective absorption of Ca^{2+} in leaves, thereby enhancing the selective transport of Ca^{2+} from root to shoot to maintain the low cell osmotic potential and the stability of the cell membrane. In addition, studies have shown that the increase in intracellular Ca^{2+} contents under salt stress can inhibit the outflow of K^+, thereby alleviating the damage of salt stress to plants [57]. Therefore, the upward transportation of Ca^{2+} in the roots of *Salix alba*. may be an important mechanism for it to maintain the balance of K^+ and Na^+ in the aerial part, establish ion homeostasis in the aerial part, and adapt to salt stress. However, due to the limited ability of the roots of *Salix alba*. to absorb Ca^{2+}, under high salt stress, the absorption of the roots will not be able to offset the loss of nutrient elements caused by ion poisoning.

4.3 Effects of salt stress on photosynthetic parameters of *Salix alba*

Photosynthesis is a key metabolic process that provides material energy for plants. High salt stress will comprehensively affect the photosynthesis of plants through osmotic stress, ion toxicity, and feedback inhibition caused by the accumulation of photosynthetic products [58]. These effects will cause the destruction of the membrane structure and the imbalance of ions in tissue cells, affecting the absorption of light energy by plants and the process of carbon assimilation [59]. This change inhibits the formation of leaf primordia and reduces the photosynthetic area and carbon assimilation of individual plants, resulting in physiological metabolic disorders and the accumulation of toxic substances. In fact, the energy supply related to photosynthesis, carbohydrate metabolism, and the TCA cycle are all inhibited by salt stress [60].

Because stomata are directly connected to the external environment, their coordinated response under stress determines whether the photosynthetic capacity of the plant is normal [61]. In this experiment, the P_n, E, and g_s did not change significantly when the NaCl concentration was 171mM/L. As the salt concentration further increased, each index decreased significantly, which is basically consistent with the results of previous studies [62,63]. When the NaCl concentration was less than 342mM/L, the C_i of the *Salix alba*. leaves decreased with decreasing g_s. Thus, the diffusion resistance of CO_2 in the leaves increases, and the carbon sequestration ability weakens. The stoma factor is the dominant factor restricting the decline in *Salix alba*. leaf photosynthesis. Later, as the degree of salt stress further intensified, the C_i increased with the decreasing g_s, and the photosynthetic system activity of the mesophyll cells

decreased, resulting in a decrease in the assimilation capacity, which is a typical non-stomatal limiting factor. Previous studies have shown that under adverse stress, stomatal restriction and non-stomatal restriction and the interaction of the two will reduce the photosynthetic rate of plants; under mild stress, stomatal restriction is dominant; and under severe stress, stomatal restriction leads to non-stomatal restriction [64,65]. Our experiment also supports this view.

4.4 Effects of salt stress on chlorophyll fluorescence kinetics of *Salix alba*

The OJIP curve contains a great deal of information about the original photochemical reaction of the PSII reaction center [66]. When environmental conditions change, chlorophyll fluorescence can directly or indirectly affect the photosystem performance of plants [67]. The changes in the PSII can reflect the impact of changes in the stress environment on the photosynthetic capacity of plants and the adaptation mechanism of photosynthetic machinery to environmental changes. High salt stress can inhibit or destroy parts of the functions of PS II, hinder the original photochemical reaction and electron transfer process of PS II, and reduce the photosynthetic capacity of *Salix alba*. leaves. This consequence may be the result of the accumulation of Na^+. The typical fast fluorescence kinetics curve generally has O, J, I, and P phases during the rising phase of fluorescence [68]. This study shows that when the concentration of NaCl is ≥ 342mM/L, the OJIP curve of *Salix alba*. will be deformed to OKJIP, the fluorescence values of points I and P will decrease significantly, and obvious inflection point K will appear. The occurrence of the K point is caused by damage to the PSII donor side oxygen release complex (OEC) due to the inhibition of the water lysis system and the receptor-side part before Q_A, and the relatively variable fluorescence of the K point can represent the degree of OEC damage [69,70]. In addition, the high salt treatment greatly shortened the time required to reach the P point (the maximum fluorescence value). This result indicates that the higher the degree of salt stress, the greater the damage to the stability of the PS II reaction center and the OEC on the PS II donor side of *Salix alba*. leaves, the weaker the ability to provide electrons downstream and the stronger the reduction of the PS II acceptor side is hindered.

The φPo, Ψo, φEo, φDo reflect the energy distribution ratio of plants. In this study, when the NaCl concentration was 171mM/L, there was no significant difference among the indicators. As the stress intensified, the φPo, Ψo and φEo decreased significantly while the φDo increased significantly, which is different from the results of Huang Qinqin et al. [71]. This finding shows that *Salix alba*. adjusted the energy distribution ratio of the PSII reaction center under different degrees of stress. This adjustment occurs to increase the quantum ratio used for heat dissipation and

reduce the proportion of energy in photochemical reactions, which is an adaptive regulation mechanism of *Salix alba*. under salt stress. The decrease in the φPo, Ψo and φEo indicates that the photosynthetic machinery is clearly damaged, the ability to reduce the Q_B and P_Q on the PSII receptor side is diminished, and the electron transfer process is inhibited. Plants are prone to occur or aggravate photoinhibition in adverse environments [69]. In this study, when the concentration of NaCl was greater than 171mM/L, the PI_{ABS}, PI_{CSm} and DF_{CSm} all showed a significant downward trend. This trend shows that *Salix alba*. leaves exhibit photoinhibition, the PSII reaction center is reversibly inactivated or irreversibly degraded, the conversion efficiency of light energy is reduced, and the function of the photosynthetic apparatus is impaired, which restricts the normal progress of photosynthesis.

In this study, 171mM/L NaCl stress had no significant effect on the growth status of the Salix alba. root system, ion distribution or photosynthetic fluorescence characteristics and even increased these parameters to a certain extent. As the salt treatment concentration gradually increased, the average root number, average root length, and rooting index decreased significantly; Na^+ accumulated in the root system, K^+ and Ca^{2+} were significantly lost; the photosynthetic rate decreased significantly, the PS II reaction center was partially inactivated, and the donor side OEC and the electron acceptor on the acceptor side were damaged. *Salix alba* can respond to salt stress by intercepting Na^+ in the root system, improving the selective absorption of K^+ and Ca^{2+} and the ground transportation capacity, and increasing the quantum ratio used for heat dissipation, indicating that *Salix* willow has some tolerance to salt stress environments.

Supporting Information

S1 Table. Effects of salt stress on the contents of three ions in roots and leaves of *Salix alba L*

Acknowledgments

We express sincere gratitude to all the authors involved in this study.

Author Contributions

Conceptualization: Xin Ran, Xiao Wang

Data curation: Xin Ran, Xiao Wang, Xiaokuan Gao, Haiyong Liang, Bingxiang Liu, Xiaoxi Huang

Formal analysis: Xiao Wang, Xin Ran, Bingxiang Liu,

Funding acquisition: Haiyong Liang, Bingxiang Liu

Methodology: , Xiaokuan Gao, Bingxiang Liu

Resources: Bingxiang Liu, Xiao Wang

Writing-original draft: Xiao Wang, Bingxiang Liu

Writing-review&editing: Xiaokuan Gao, Haiyong Liang, Bingxiang Liu

References

1. ZHANG J L, FLOWERS T J, WANG S M (2010) Mechanisms of sodium uptake by roots of higher plants. Plant and Soil 326(1-2): 45-60.
2. MA W Y (2004) Progress in research of plant tolerance to Saline Stress. Agriculture and Technology 24(04): 95-99. [in Chinese]
3. ZHU J F, CUI Z R, WU C H, et al (2018) Research Advances and Prospect of Saline and Alkali Land Greening in China. World Forestry Research 31(04): 70-75. [in Chinese]
4. YANG Q S, LIU D, GAO W D, et al (2016) Research Progress on Stress Resistance of Willow. Forestry Science and Technology Newsletter (07): 24-27. [in Chinese]
5. WANG Q Z, LIU Q, GAO Y N, et al (2017) Review on the mechanisms of the response to salinity-alkalinity stress in plants. Acta Ecologica Sinica 37(16): 5565-5577. [in Chinese]
6. LI Z Y (2017) Physiological responses of Salix psammophila seedlings to complex saline-alkali stress. Beijing: Chinese Academy of Forestry sciences. [in Chinese]
7. LIU Y M, CHENG C, JIANG L, et al (2019) Comparisons on growth, salt ion distribution, and relative expression of SOS1 gene in three species of Tamarix Linn. under NaCl stress. Journal of Plant Resources and Environment 28(01): 1-9. [in Chinese]

8. ZHANG X, HE K N, SHI C Q, et al (2017) Effects of salt stress on growth and physiological characteristics of Tamarix chinensis and Nitraria tangutorum seedlings. Journal of Northwest A & F University (Natural Science Edition) 45(01): 105-111. [in Chinese]

9. LI Z Y, CONG R C, YANG Q S, et al (2017) Effects of saline-alkali stress on growth and osmotic adjustment substances in willow seedlings. Acta Ecologica Sinica 37(24): 8511-8517. [in Chinese]

10. ZHANG J L, LI H R, GUO S Y, et al (2015) Research advances in higher plant adaptation to salt stress. Acta Prataculturae Sinica 24(12): 220-236. [in Chinese]

11. YANG S, LIU Z X, ZHANG H X, et al (2013) Comprehensive evaluation of salt tolerance and screening identification indexes for three tree species. Scientia Silvae Sinicae 49(1): 91-98. [in Chinese]

12. RAUT R, SHARMA S, BAJRACHARYA R M. (2012) Biotic response to acidification of lakes: a review. Kathmandu University Journal of Science, Engineering and Technology 8(1): 171-184.

13. KALAJI H M, JAJOO A, OUKARROUM A, et al (2016) Chlorophyll a fluorescence as a tool to monitor physiological status of plants under abiotic stress conditions. Acta Physiologiae Plantarum 38(4): 1-11.

14. Kolozsvári Ildikó,Kun Ágnes,Jancsó Mihály, et al (2021) Utilization of Fish Farm Effluent for Irrigation Short Rotation Willow (Salix alba L.) under Lysimeter Conditions. Forests 12(4).

15. Barnes J, Anderson LA and Phillipson JD. (2007) Herbal Medicines.London. Chicago: Pharmaceutical Press :598-600 .

16. HUI Y H, WANG R C.(2004) Determination of salicin in the extract of Salix officinalis by RP-HPLC. Chinese Traditional and Herbal Medicines (05):48-49.[in Chinese]

17. KONG J H, WANG Y F, YANG X F, et al (2012) Extraction of salicin from populus euphratica leaves by alcohol-alkali method . Food science and technology 37(11):205-209.[in Chinese]

18. DUAN H, ZHAI K F, GAO G Z, et al (2012) Purification of salicin from the roots of maple leaf by macroporous adsorption resin. Food science 33(22):99-102.[in Chinese]

19. Mataruga Z , S Jarić, M Marković, et al (2020) Evaluation of Salix alba, Juglans regia and Populus nigra as

biomonitors of PTEs in the riparian soils of the Sava River. Environmental Monitoring and Assessment 192(2):131.

20. Bajraktari Demush,Petrovska Biljana Bauer, Zeneli Lulzim, et al (2020) Soil chemical evaluation and power plant ash impact on chemical properties of Salix alba L. (Fam. Salicaceae): The impact of bioaccumulation. Toxicology Research and Application 4(4).

21. Regni Luca,Bartucca Maria Luce,Pannacci Euro, et al (2021) Phytodepuration of Nitrate Contaminated Water Using Four Different Tree Species.Plants (Basel, Switzerland) 10(3).

22. A. S. Quiñones Martorello,M. E. Fernάndez,M.G. Monterubbianesi, et al (2020) Effect of combined stress (salinity + hypoxia) and auxin rooting hormone addition on morphology and growth traits in six Salix spp. clones. New Forests: International Journal on the Biology, Biotechnology, and Management of Afforestation and Reforestation 51(1).

23. HU A S, ZHANG X D, GUO W J, et al(2021) Ion Absorption, transport and distribution of Malus petalis under salt Stress. Plant Physiology Journal 57(09):1829-1838. [in Chinese]

24. LI B B, OU YJ, WANG J Y, et al(2017) Effects of NaCl on growth, development and some physiological characteristics of salix matsulosa. Journal of tianjin normal university (natural science edition) 37(06):37-42. [in Chinese]

25. CHEN, T,White,James F, (2021) Exogenous spermidine enhances Epichloë endophyte-induced tolerance to NaCl stress in wild barley (Hordeum brevisubulatum). Plant and Soil(prepublish).

26. YANG S (2010) Study on selection and evaluation criteria of salinity-tolerance tree species in coastal region. Beijing: Chinese Academy of Forestry sciences. [in Chinese]

27. YU B J, LUO Q Y, CAO A Z, et al (2001) Comparison of salt tolerance and ion effect in cultivated and wild soybean. Journal of Plant Resources and Environment 10(01): 25-29. [in Chinese]

28. ZHENG Q S, WANG R L, LIU Y L (2001) Effects of Ca^{2+} on Absorption and Distribution of Ions in Salt-treated Cotton Seedlings. Plant Physiology Journal 27(4): 325-330. [in Chinese]

29. YANG X Y, ZHANG W H, WANG Q Y, et al (2003) Salt tolerance of wild soybeans in Jiangsu and its relation

with ionic distribution and selective transportation. Chinese Journal of Applied Ecology 14(12): 2237-2240. [in Chinese]

30. STRASSER R J, SRIVASTAVA A, TSIMILLI-MICHAEL M (2000) The fluorescence transient as a tool to characterize and screen photosynthetic samples. In: Yunus M, Pathre U, Mohanty P (eds). Probing Photosynthesis: Mechanism, Regulationand Adaptation. London: Taylor and Francis Press, 445–483.

31. STRASSER B J, STRASSER R J (1995) Measuring fast fluorescence transients to address environmental questions: The JIP test. In: Mathis P ed. Photosynthesis: From Light to Biosphere. Dordrecht, the Netherlands: KAP Press 5: 977~980.

32. SUN J, JIA Y X, GUO S R, et al. (2010) Resistance of spinach plants to seawater stress is correlated with higher activity of xanthophyll cycle and better maintenance of the chlorophyll metabolism. Photosynthetica 48(4): 567-579.

33. YANG Z Q, QIU Y X, LIU Z X, et al (2016) Effects of soil water stress on root and overground growth of plant tomato. Acta Ecologica Sinica 36(3): 748-757. [in Chinese]

34. WANG S F, HU Y X, SUN H J, et al (2014) Effects of salt stress on growth and root development of two oak seedlings. Acta Ecologica Sinica 34(4): 1021-1029. [in Chinese]

35. CI D W, ZHANG Z M, DING H, et al (2015) Evaluation and selection indices of salinity tolerance in peanut seedling[J]. Acta Ecologica Sinica 35(03): 805-814. [in Chinese]

36. WANG S F, HU Y X, LI Z L, et al (2010) Effects of NaCl stress on growth and mineral ion uptake, transportation and distribution of Quercus virginiana. Acta Ecologica Sinica 30(17): 4609-4616. [in Chinese]

37. CHEN X F,ZHANG R D,XINNG Y F, et al (2021) The efficacy of different seed priming agents for promoting sorghum germination under salt stress. PloS one 16(1)

38. ZHU J J, MA H J, QIN H Y. et al (2021) Effects of potassium solubilizing bacteria and salt stress on root characteristics and physiological metabolism of Lycium barbarum seedlings. Agricultural Research in the Arid Areas 39(05):50-58+65.[in Chinese]

39. LIU S H, ZHU X S, YAN M, et al (2020) Effects of NaCl immersion on root growth characteristics of hybrid

rice seedlings under salt stress. Journal of southwest university (natural science edition)42(08):59-65.[in Chinese]

40. Iqbal M, Ashraf M, Jamil A, et al (2006) Does seed priming induce changes in the levels of some endogenous plant hormones in hexaploid wheat plants under salt stress. Journal of Integrative Plant Biology 48(2): 181 – 189.

41. Tsegay BA, Yohannes G (2013) The role of seed priming in improving seedling growth of maize (Zea mays L.) under salt stress at field conditions. Agricultural Sciences 4(12): 666 – 672.

42. LI Z L (2020) Evaluation of salt tolerance of four chrysanthemum and its related genera. Liaoning Forestry Science and Technology (02):36-38. [in Chinese]

43. [2] SHU J, LIU S H, ZHANG A H, et al (2021) Effects of NaCl stress on photosynthetic characteristics, chlorophyll fluorescence and osmotic regulatory substances in Cherry seedlings. Journal of Shanxi Agricultural Sciences 49(07):834-838.[in Chinese]

44. LIU X W, XIA B, CHEN B, et al (2021) Effects of salt Stress on photosynthetic physiology of Chrysanthemum and Chrysanthemum shennong and their F_1 hybrids. Journal of Northeast Forestry University 49(05):32-39.[in Chinese]

45. LUO D, SHI Y J, SONG F H, et al (2019) Effects of salt stress on growth, photosynthetic and fluorescence characteristics and root architecture of heterosis hazel seedlings. Chinese journal of applied ecology 30(10):3376-3384.[in Chinese]

46. RUIZ K B, BIONDI S, MARTINEZ E A, et al (2016) Quinoa – a model crop for understanding salt-tolerance mechanisms in halophytes. Plant Biosystems 150(2): 357-371.

47. HAO H, CAO L, CHEN W N, et al (2020) Effects of salt stress on the ion balance and physiological-biochemical characteristics of Quercus dentata seedlings. Acta Ecologica Sinica 40(19): 6897-6904. [in Chinese]

48. WANG N, QIAN W, LIU X, et al (2017) Relative contribution of Na^+/ K^+ homeostasis, photochemical efficiency and antioxidant defense system to different salt tolerance in cotton (Gossypium hirsutum L.) cultivars. Plant Physiology and Biochemistry 119: 121-131.

49. GUO P, WEI H X, ZHANG W J, et al (2016) Physiological responses of alfalfa to high-level salt stress: root ion

flux and stomatal characteristics. International Journal of Agriculture and Biology 18(1): 125-133.

50. SINGH V, SINGH A P, BHADORIA J, et al (2018) Differential expression of salt-responsive genes to salinity stress in salt-tolerant and salt-sensitive rice (Oryza sativa L.) at seedling stage. Protoplasma 255(6): 1667-1681.

51. ZHOU Q, ZHU Z L (2015) Effects of NaCl stress on seedling growth and mineral ions uptake, distribution and transportation of two varieties of Carpinus L. Journal of Beijing Forestry University 37(12): 7-16. [in Chinese]

52. ZHU J K (2003) Regulation of ion homeostasis under salt stress. Current Opinion in Plant Biology 6(5): 441-445.

53. DONG F, CAO J, LI X T, et al (2016) Effects of various types of salt stress on ion absorption, accumulation and transportation in pea (Pisum sativum) seedlings. Acta Prataculturae Sinice, 25(11): 66-75. [in Chinese]

54. JIANAER.A, YANG C W, SHI D C, et al (2007) Physiological Response of an Alkali Resistant Halophyte Kochia sieversiana to Salt and Alkali Stresses. Acta Botanica Boreali-Occidentalia Sinica 27(1): 79-84. [in Chinese]

55. ZHAO K F (1997) halophyte. Chinese Bulletin of Botany (4): 2-13. [in Chinese]

56. JIA Y, XIANG Y F, WANG L L, et al (2020) Effects of salt stress on the growth and physiological characteristics of Primula forbesii. Acta Prataculturae Sinice 29(10): 119-128. [in Chinese]

57. HAN Y X, CHEN S L, ZHANG Y H, et al (2013) Exogenous hydrogen peroxide, nitric oxide and calcium mediate root ion fluxes in two non-secretor mangrove species subiected to NaCl stress. Tree Physiology 33(1): 81-95.

58. QI Q, MA S R, XU W D (2020) Research progress on the effects of salt stress on plant growth and the physiological mechanism of salt tolerance. Molecular plant breeding 18(8): 2741-2746. [in Chinese]

59. ZHANG R J, REN F, BAI Y B, et al (2012) Research progress on the influence of stress on PSII based on the dynamic analysis of fast chlorophyll fluorescence induction. Anhui Agricultural Science 40(7): 3858-3859, 3864. [in Chinese]

60. PANG Q Y, ZHANG A Q, ZANG W, et al (2016) Integrated proteomics and metabolomics for dissecting the mechanism of global responses to salt and alkali stress in Suaeda corniculate. Plant and Soil 402(1/2): 379-394.

61. WANG Z Y, YANG Y, HUA J F, et al (2020) Effects of alkali treatment on photosynthetic characteristics and physiology of seedling leaves of Chinese fir 406. Journal of Plant Resources and Environment 29(04): 72-74. [in Chinese]

62. LI X F, NI Z M, WU Y Y, et al (2015) Effects of salt stress on photosynthetic characteristics and leaf cell structure of ‘Yinhong’ grape seedlings. Acta Ecologica Sinica 35(13): 4436-4444. [in Chinese]

63. YANG J Y, ZHENG W, TIAN Y, et al (2011) Effects of various mixed salt-alkaline stresses on growth，photosynthesis， and photosynthetic pigment concentrations of Medicago ruthenica seedlings. Photosynthetica 49(2): 275-284.

64. LI X X, LIU B X, GUO Z T, et al (2013) Changes of photosynthetic characteristics and fast chlorophyll fluorescence induced kinetic curve of rhizoma coptidis leaves under NaCl stress. Chinese Journal of Applied Ecology 24(9): 2479－2484. [in Chinese]

65. ZHANG H H, ZHANG X L, LI X, et al (2012) Effects of NaCl and Na_2CO_3 stress on growth and photosynthetic characteristics of mulberry seedlings. Chinese Journal of Applied Ecology 23(3): 625－631.

66. LI P M, GAO H Y (2005) Application of fast chlorophyll fluorescence induction kinetics analysis in photosynthesis. Journal of Plant Physiology and Molecular Biology 31(6): 559 -566. [in Chinese]

67. KONG F, WANG W C, HU H, et al (2019) Effects of NaCl Treatment on Photosynthetic and Fast Chlorophyll Fluorescence Characteristics in Herbaceous Peony. Molecular Plant Breeding 17(22): 7531-7537. [in Chinese]

68. HUANG Q X, ZHAO S, LIU C M, et al (2015) Effects of Shading Treatments on Chlorophyll Fluorescence Characteristics of Sabina vulgaris Seedlings Grown in Iron Tailings Media. Scientia Silvae Sinicae 51(6): 17-26. [in Chinese]

69. KONG F, LIU X Y, WANG G Z, et al (2016) Effect of fertilizer application on photosynthesis and fast chlorophyll fluorescence characteristics of walnut. Forestry scientific research 29(5): 764-769. [in Chinese]

70. LI H, LI H W, LV Y J, et al (2019) Salt priming protects photosynthetic electron transport against low-temperature-induced damage in wheat. Sensors 20(1): 62.

71. HUANG Q Q, YANG Z Q, LI J S, et al (2019) Effect of Water and Nitrogen Coupling on Rapid Fluorescence Induction Kinetics Characteristics of Facility Grape Leaves. Chinese Journal of Agrometeorology 40(9): 557-573. [in Chinese]

Figur captions:

Fig.1 Experimental scenes of the effects of salt stress on *Salix alba L*

Fig. 2 Changes of root growth of *Salix alba L* under salt stress

Fig. 3 Effects of salt stress on root growth of *Salix alba* cuttings.

Fig. 4 Effects of salt stress on ion content in roots and leaves of *Salix alba L*

Fig. 5 Effects of salt stress on Na^{+}/ Ca^{2+} in roots and leaves of *Salix alba L*

Fig. 6 Effects of salt stress on Na^{+}/ K^{+} in roots and leaves of *Salix alba L*

Fig. 7 Effects of salt stress on selective uptake and transportation of ion in roots and leaves of *Salix alba L*.

Fig. 8 Effects of salt stress on photosynthetic parameter (Pn and E)in leaves of *Salix alba L*

Fig. 9 Effects of salt stress on photosynthetic parameter (Ci and gs) in leaves of *Salix alba L*

Fig. 10 Effect of salt stress on the fast induction curves of chlorophyll a fluorescence（O-J-I-P curve）of *Salix alba L* leave

Fig. 11 changes of Chlorophyll a fluorescence parameters under salt stress of *Salix alba L*

Fig. 12 The changes of Performance index and driving force (PIABS and DFCSm)under different salt stress

Fig. 13 The changes of Performance index and driving force (PICSm)under different salt stress

Figure5
Click here to access/download;Figure;figure5.eps

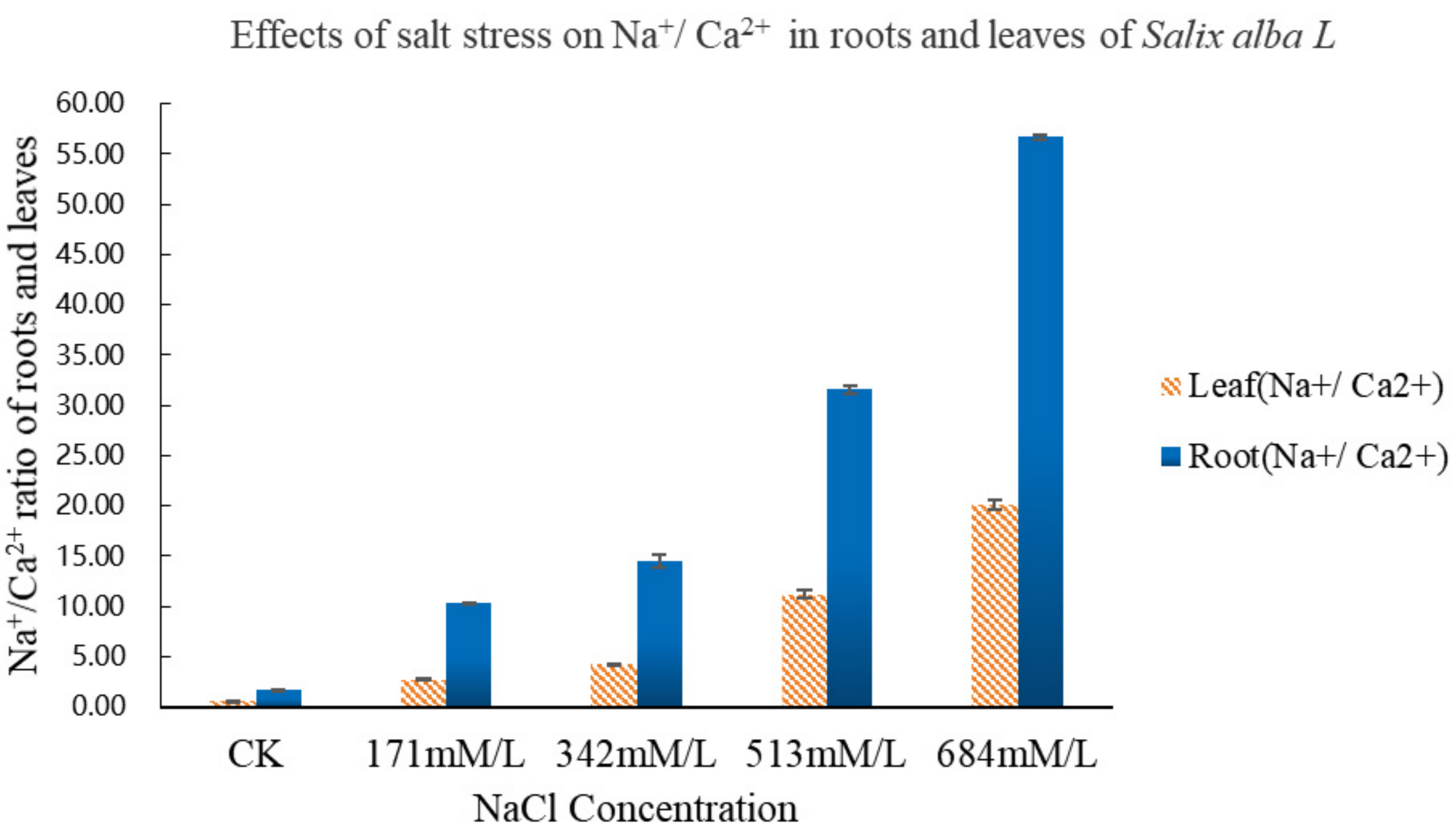

Figure6

Click here to access/download;Figure;figure6.eps

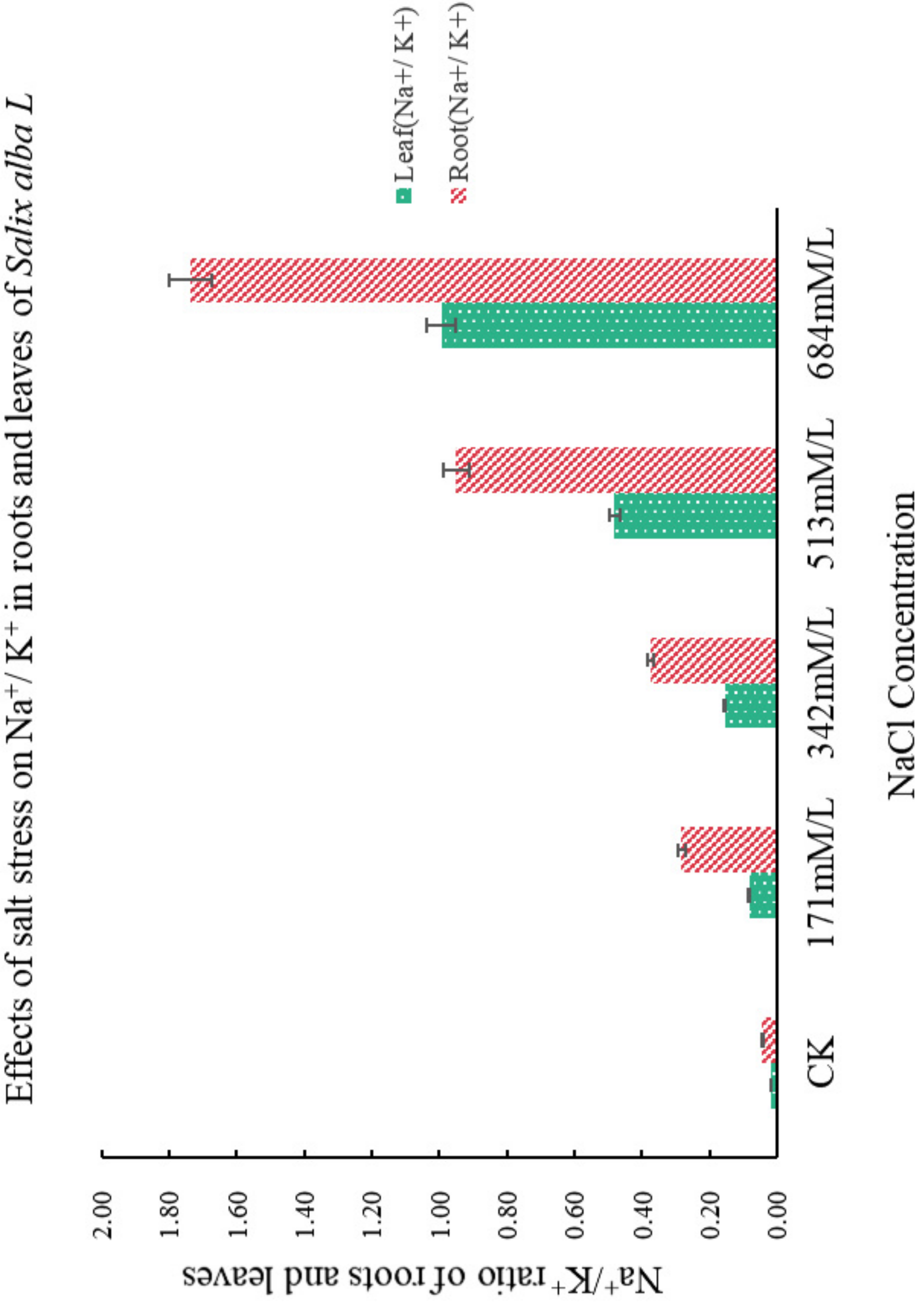

Figure7

Click here to access/download;Figure;figure7.eps

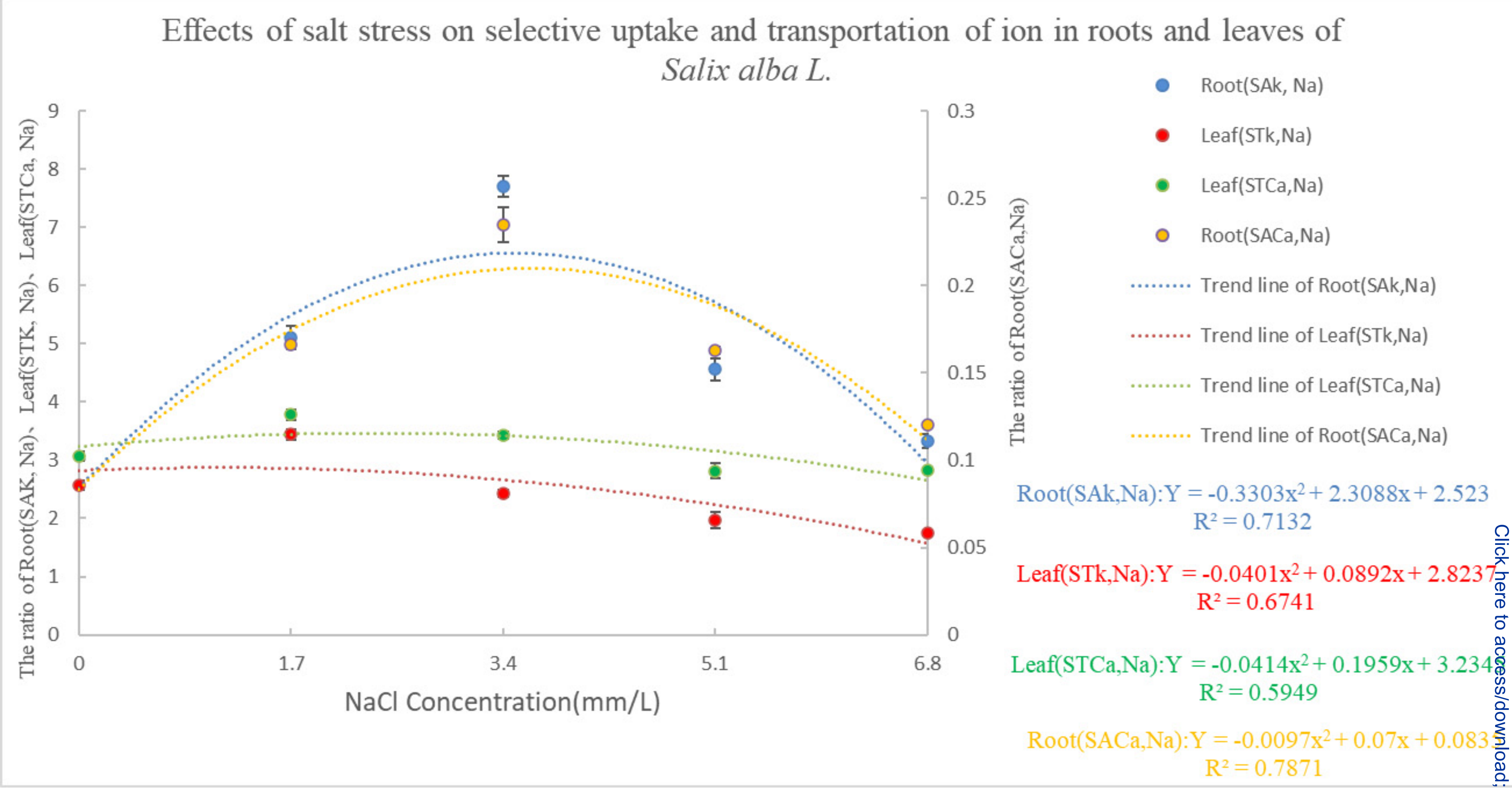

Figure8
Click here to access/download;Figure;figure8.eps

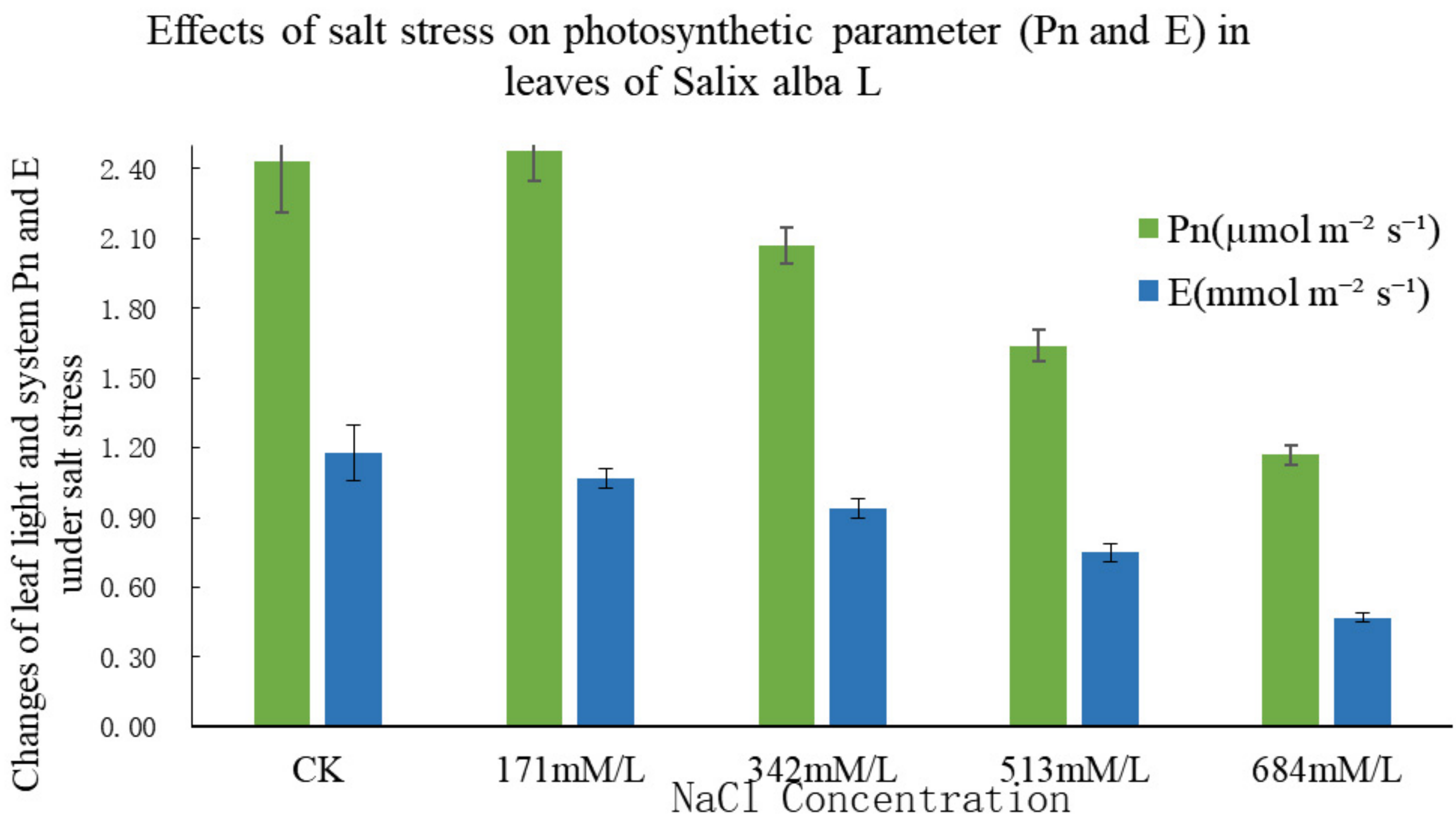

Click here to access/download;Figure;figure9.eps

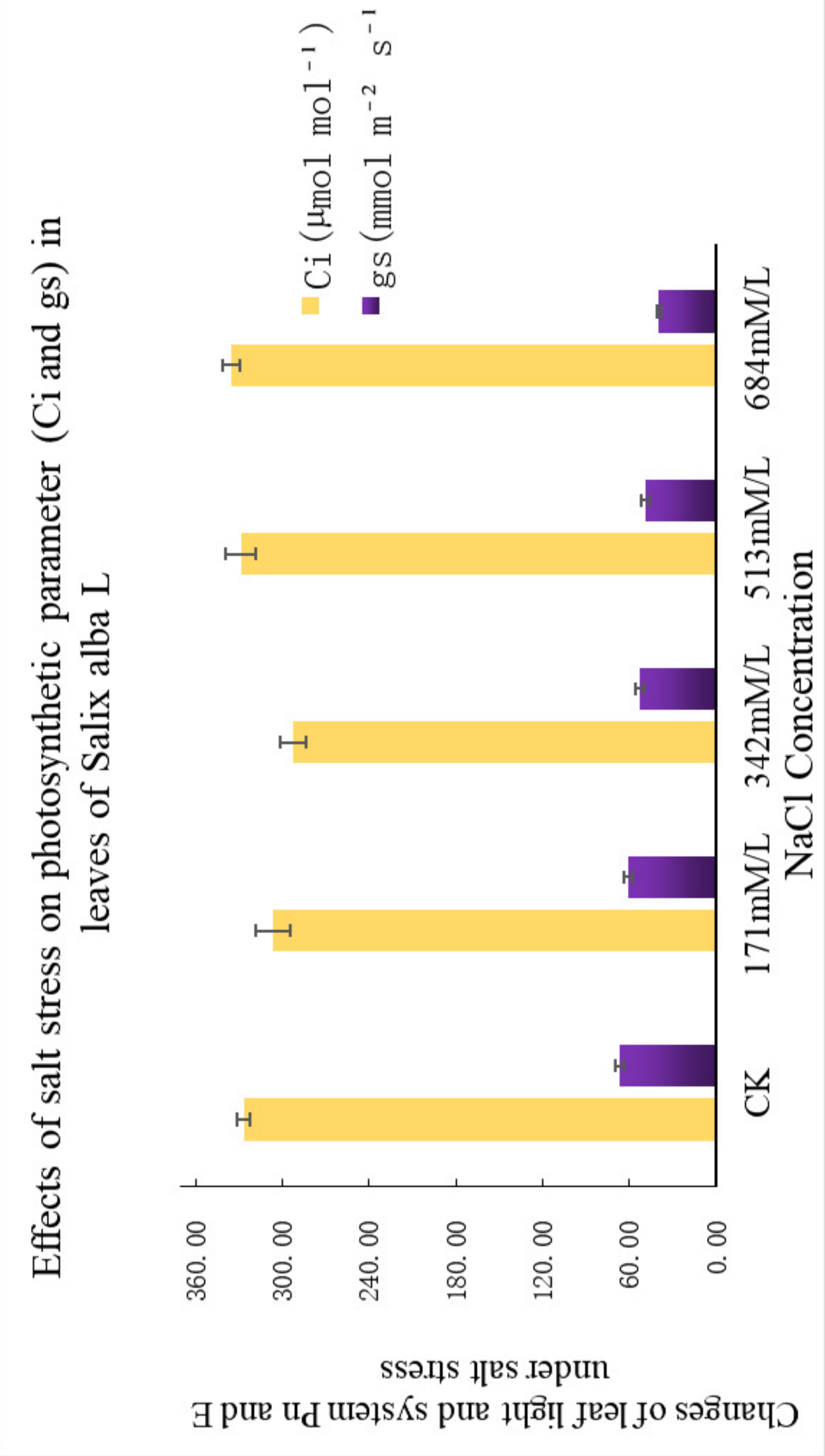

Figure10 Click here to access/download;Figure;figure10.eps

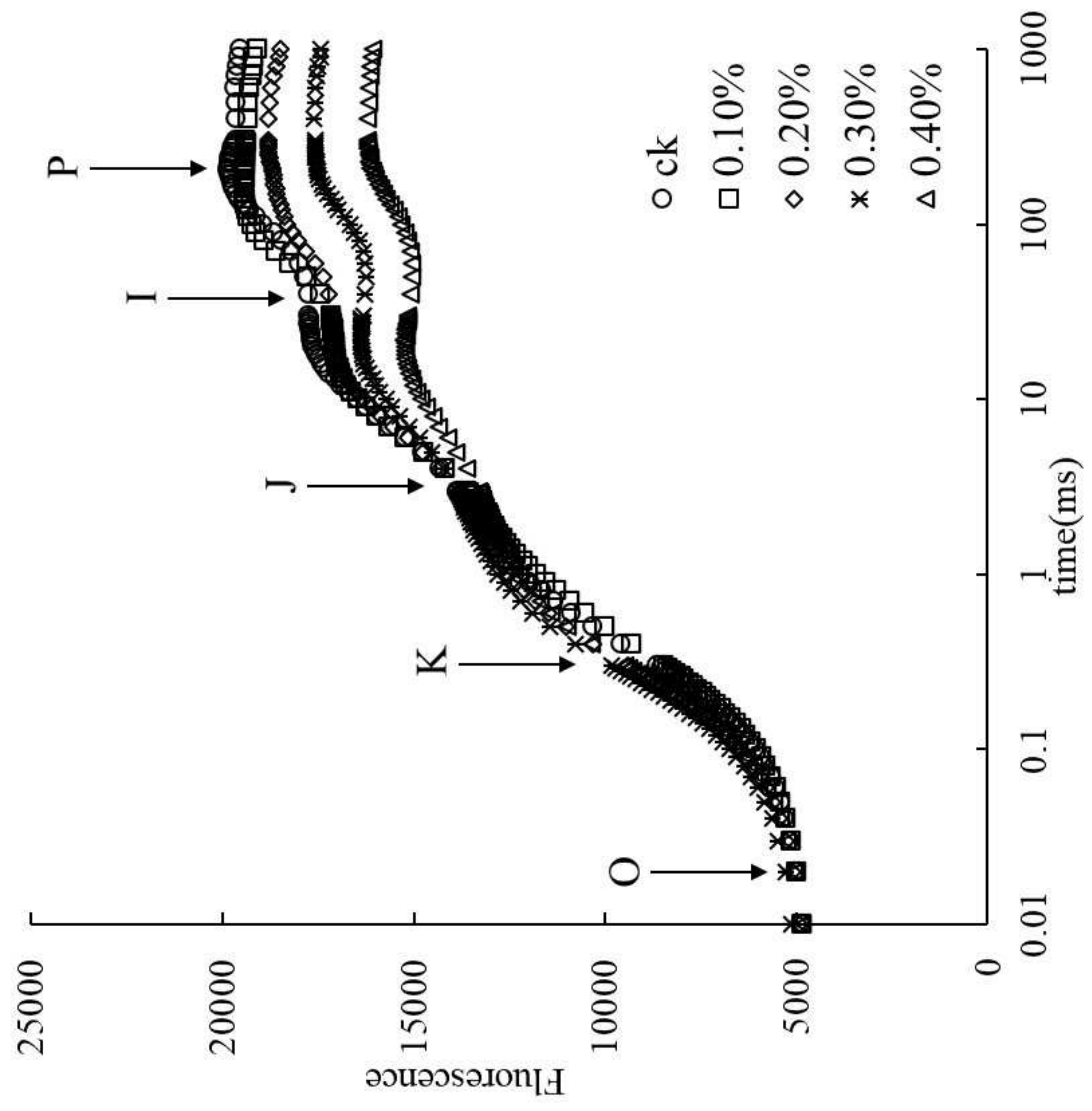

Figure11

Click here to access/download;Figure;figure11.eps

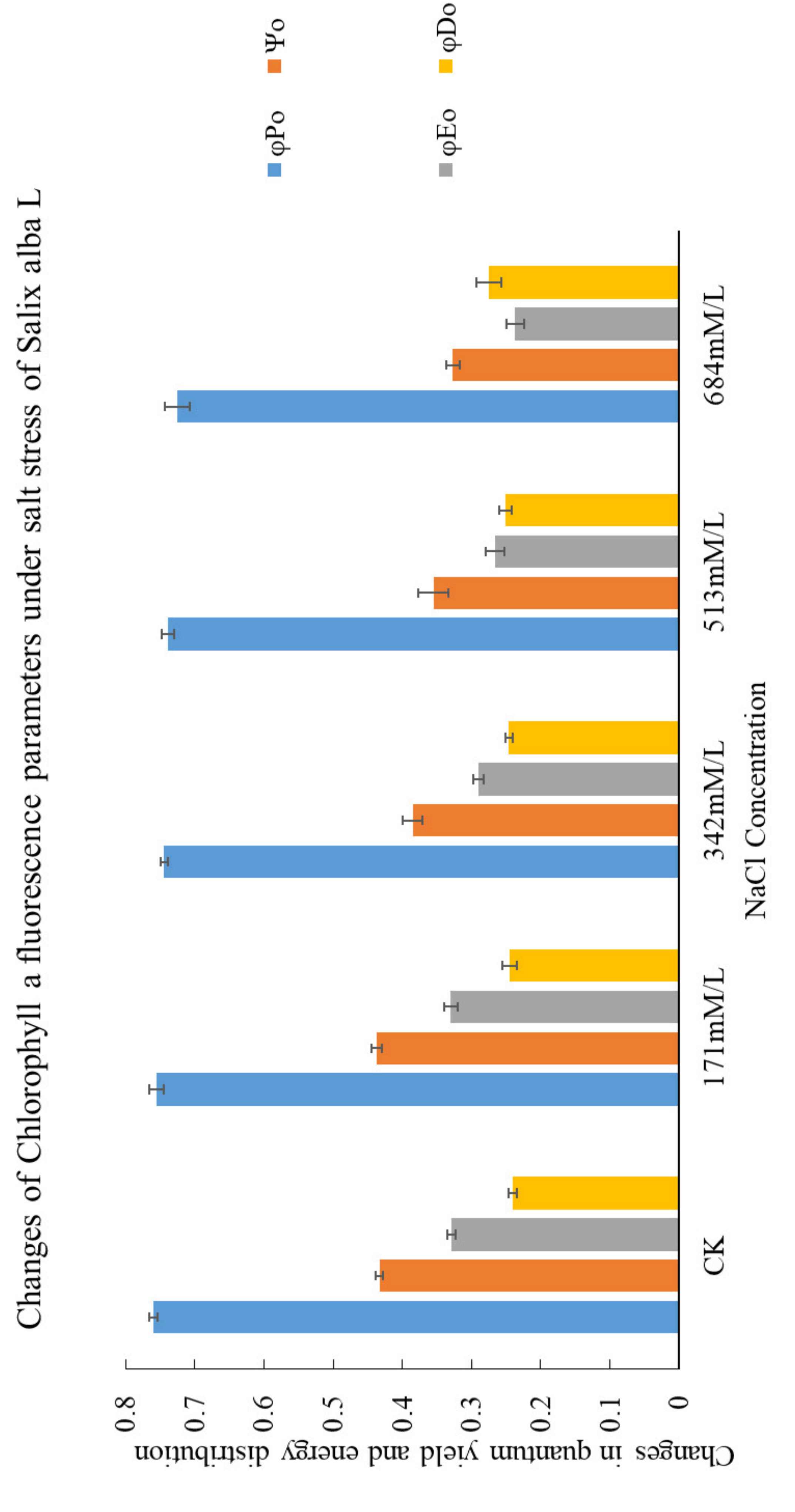

Figure12 Click here to access/download;Figure;figure12.eps

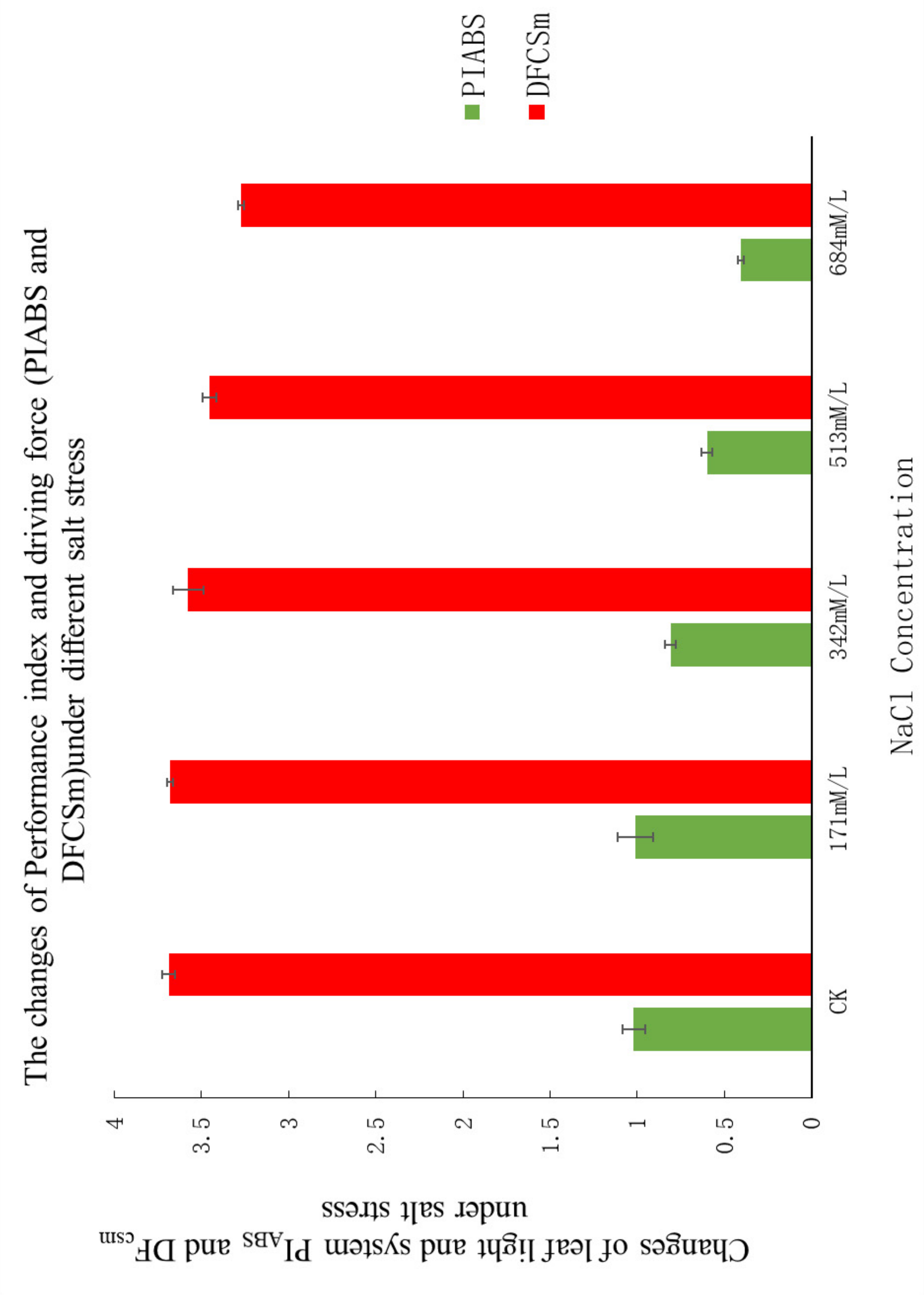

Figure13

Click here to access/download;Figure;figure13.eps

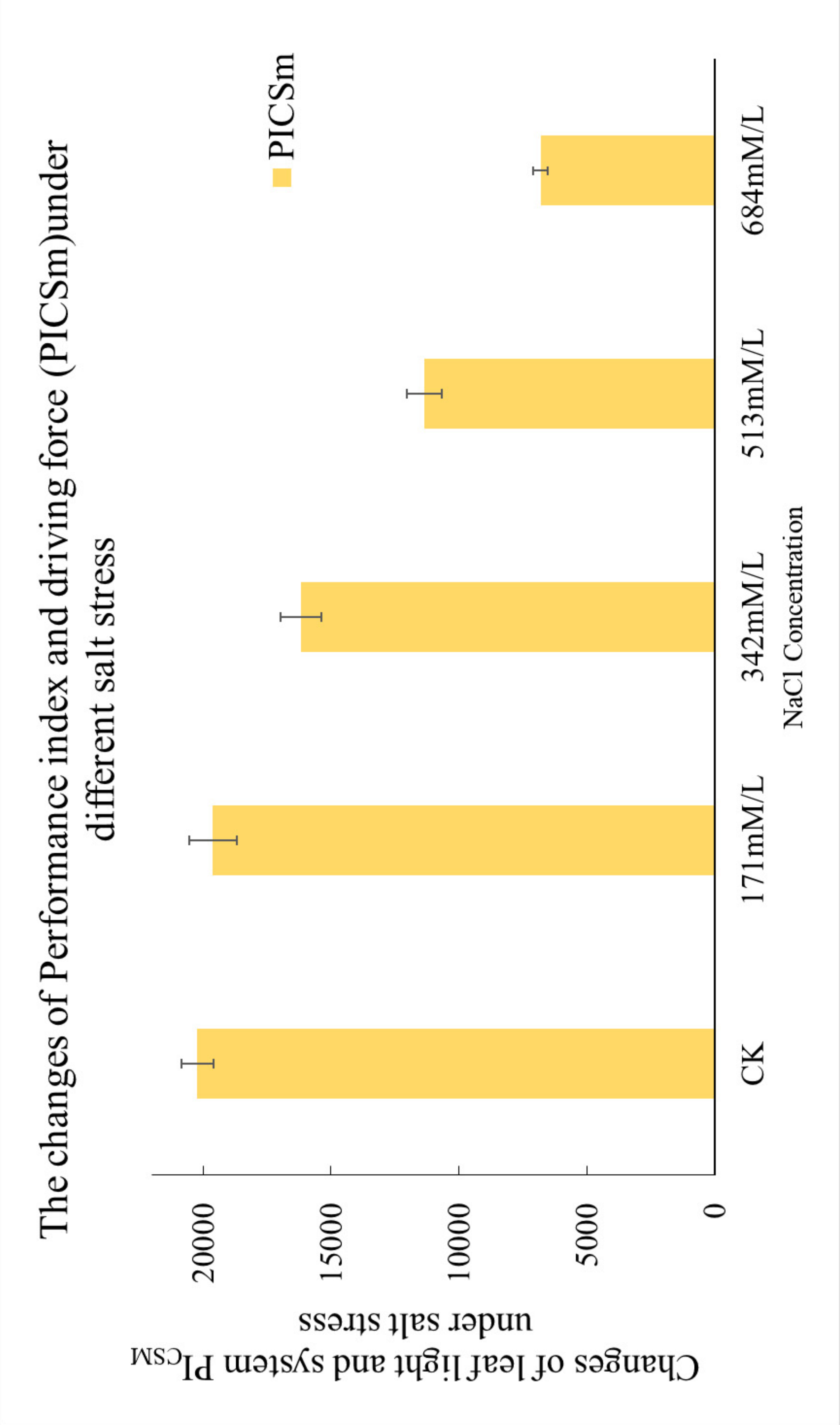

Figure1

Click here to access/download;Figure;figure1.eps

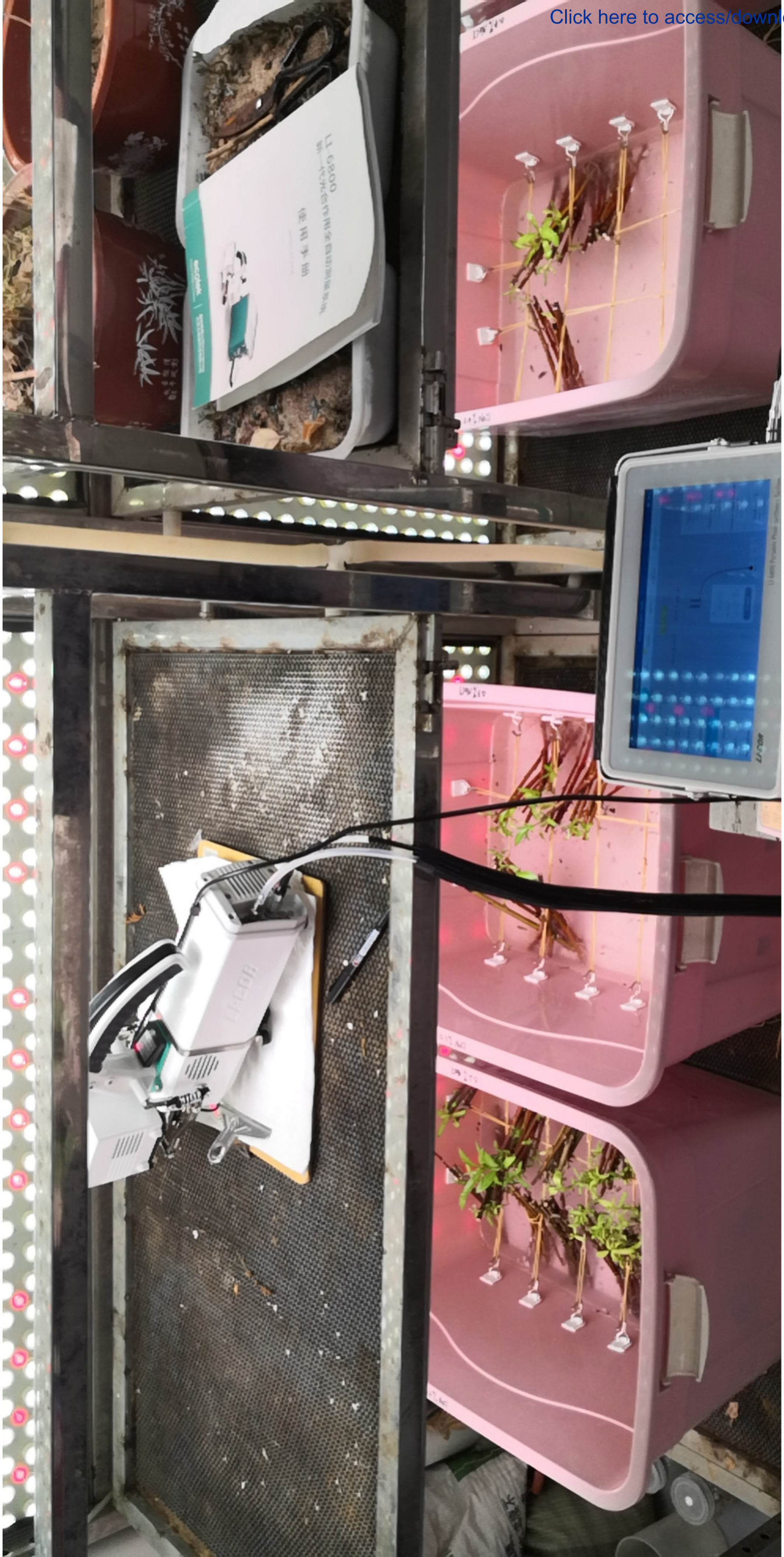

Experimental scenes of the effects of salt stress on Salix alba L

Click here to access/download;Figure;figure2.eps

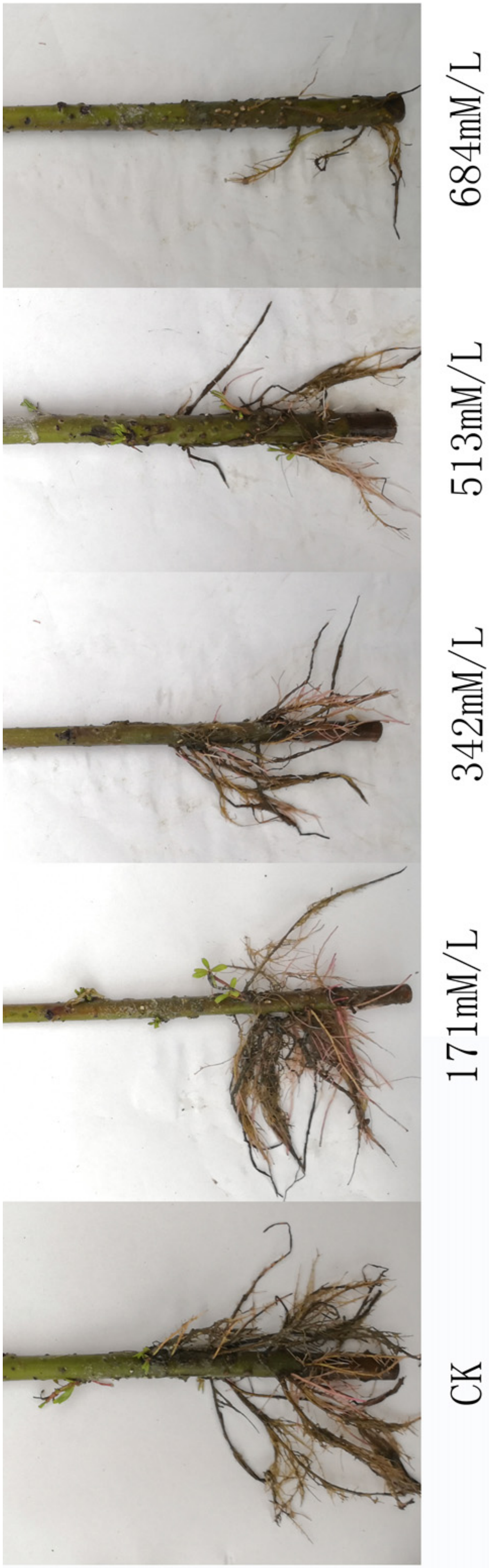

Changes of root growth of Salix alba under salt stress

Figure3

Click here to access/download;Figure;figure3.eps

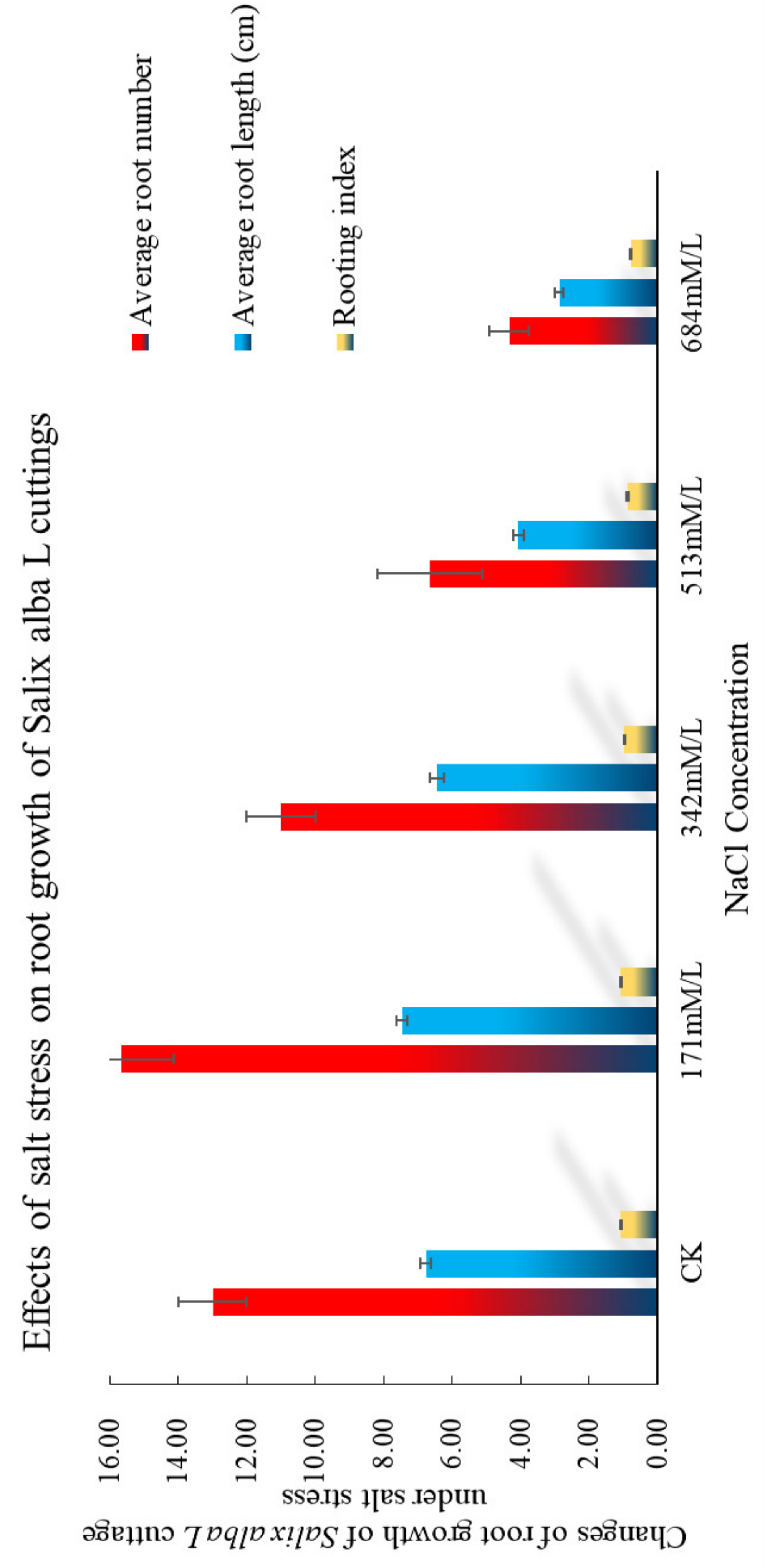

Figure4 Click here to access/download;Figure;figure4.eps

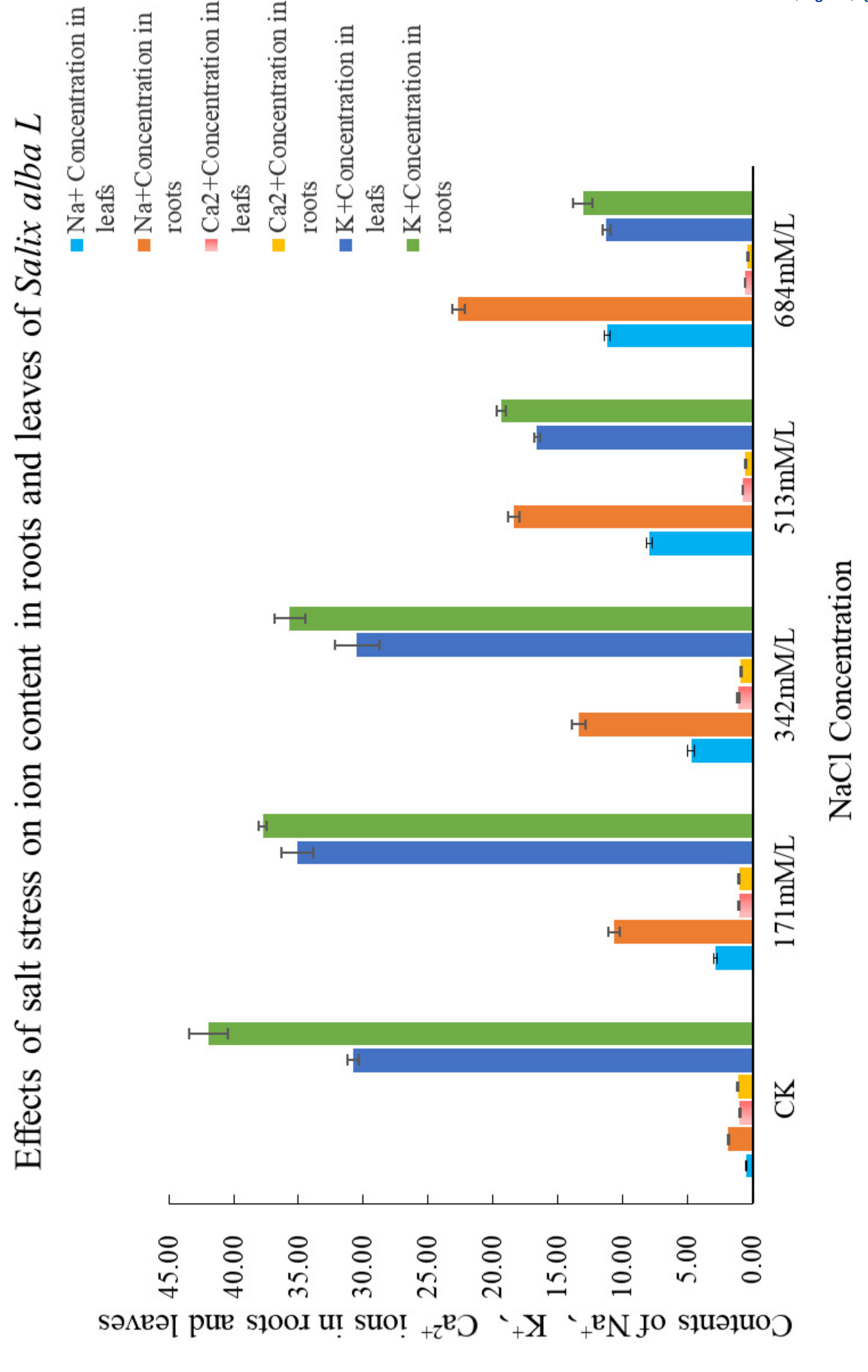

Click here to access/download
Supporting Information
S1 Appendix.xlsx

NOTES: RED means DELETE

BLUE means changes based on the editors' suggestions and some we made

BLACK means the original

Effects of salt stress on the photosynthetic physiology and mineral ion absorption and distribution in white willow (*Salix alba* L)

Xin Ran[1], Xiao Wang[1], Xiaokuan Gao[2], Haiyong Liang[1], Bingxiang Liu[1,3*], Xiaoxi Huang [1]

1. College of Forestry, Hebei Agricultural University , Baoding 071000, Hebei, China;

2. College of Life Science, Hengshui University, Hengshui 053000, Hebei, China;

3. Hebei Urban Forest Health Technology Innovation Center, Baoding 071000, Hebei, China

* Correspondence author.

E-mail address: proser211@126.com (Bingxiang Liu)

Abstract: [Objective] The purpose of this study was to explore the adaptive mechanism underlying the photosynthetic characteristics and the ion absorption and distribution of white willow (*Salix alba L*) in a salt stress environment in cutting seedlings. The results lay a foundation for further understanding the distribution of sodium chloride and its effect on the photosynthetic system. [Method] A salt stress environment was simulated in a hydroponics system using different NaCl concentrations in one-year-old *Salix alba*. cuttings as the test materials. The growth status of the cutting roots, ion absorption, transport and distribution in the roots and leaves, and the changes

in the photosynthetic fluorescence parameters were studied after 20 days under hydroponics. [Results] The results show that root germination and elongation are promoted in the presence of 0.1% 171mM/L NaCl, but root growth is comprehensively inhibited under increasing salt stress. Under salt stress, Na^+ accumulates significantly in the roots and leaves, and the Na^+ content and the Na^+/K^+ and Na^+/Ca^{2+} root ratios are significantly greater than those in the leaves. When the NaCl concentration is ≤ 0.2% 342mM/L, *Salix alba.* can maintain relatively stable K^+ and Ca^{2+} contents in its leaves by improving the selective absorption and accumulation of K^+ and Ca^{2+} and adjusting the transport capacity of mineral ions to aboveground parts, while K^+ and Ca^{2+} levels are clearly decreased under high salt stress. With increasing salt concentrations, the net photosynthetic rate (P_n), transpiration rate (E) and stomatal conductance (g_s) of leaves decrease gradually overall, and the intercellular CO_2 concentration (C_i) first decreases and then increases. When the NaCl concentration is < 342mM/L, the decrease in leaf P_n is primarily restricted by the stomata. When the NaCl concentration is > 342mM/L, the decrease in the P_n is largely inhibited by non-stomatal factors. Due to the salt stress environment, the OJIP curve (Rapid chlorophyll fluorescence) of *Salix alba*. turns into an OKJIP curve. When the NaCl concentration is > 171mM/L, the fluorescence values of points I and P decrease significantly, which is accompanied by a clear inflection point (K). The quantum yield and energy distribution ratio of the PS II reaction center change significantly (φPo, Ψo and φEo show an overall downward trend while φDo is promoted). The performance index and driving force (PI_{ABS}, PI_{CSm} and DF_{CSm}) decrease significantly when the NaCl concentration is > 171mM/L, indicating that salt stress causes a partial inactivation of the PSII reaction center, and the functions of the donor side and the recipient side are damaged. [Conclusion] The above results indicate that Salix alba. can respond to salt stress by intercepting Na^+ in the roots, improving the selective absorption of K^+ and Ca^{2+} and the transport capacity to the above ground parts of the plant, and increasing φDo, thus showing an ability to self-regulate and adapt.

Keywords: *Salix alba. L*; NaCl stress; root growth; ion absorption and transport; photosynthetic system characteristics; chlorophyll fluorescence kinetics

1 Introduction

With economic and social development, the problem of soil salinization has become increasingly prominent, resulting in approximately 1 billion hectares of saline-alkali land in the world[1]. The total area of China's saline-alkali land is believed to reach more than 100 million hectares[2]. Among the areas of concern, the coastal area, one of the primary types of saline-alkali land, has frequent water-salt interactions and secondary salinization because it is close to the sea[3]. Because the ecological environment of the salinization area is fragile and natural conditions are limited by many factors, it is highly significant to develop and use saline-alkali land scientifically and rationally while under pressure from a rapid population increase and a sharp decline in land resources; the goal is to advance towards the sustainable and healthy development of China's forestry and ecological environment [4].

Choosing and cultivating excellent salt-tolerant tree species through biotechnology is currently one of the most economical, effective, ecological and environmentally friendly biological measures to solve the soil salinization problem [5]. *Salix* is a deciduous tree or shrub belonging to the genus *Salix* in the Salicaceae. It has strong ecological adaptability and can grow well under saline-alkali, drought and barren soil conditions [6]. Previous studies on the salt tolerance of *Salix* plants were mostly focused on the physiological responses of seedlings to salt stress [7,8,9], but there are few studies on the adaptability of plants to salt stress specific to seedlings. High salt stress will cause plant water loss, ion imbalance and nutrient element deficiency through osmotic stress and ion poisoning [10], which will affect the normal growth and morphology of plants. A series of physiological growth changes in plants under salt stress are the comprehensive embodiment of their salt tolerance ability, among which the growth status of plant roots, the ion accumulation in different organs and the change in photosynthetic fluorescence parameters are important factors affecting the salt tolerance ability of plants [11,12,13]. These indicators can not only represent the extent of the effects of stress factors on plants, but they can also reflect the growth of plants under salt stress, the selective absorption and transport of ions, and the photosynthesis ability. Willow has the characteristics of antipyretic, analgesic, anti-inflammatory, anti-rheumatism, astringent, drought resistance[14] and anti-corrosion [15], among which the bark of White Willow contains salicin [16,17] with antibacterial, bactericidal, antioxidant, antipyretic, analgesic and other functions, and is a good natural food additive and food resource of health care products [18]. Its roots can also enrich harmful elements, reduce the impact of harmful elements on the surrounding soil [19,20], and play a role in purifying polluted water [21].*Salix alba.*has strong adaptability to adversity[22], so it has great potential for use and promotion in

the ecological management of coastal saline-alkali soil. Therefore, this experiment involved *Salix alba*. cuttings as the object and used hydroponics to simulate the seedling raising process of cuttings on coastal saline-alkali land to study the growth of their roots, the changing ion contents in the roots and leaves, and the changing photosynthetic fluorescence parameters under different salt concentrations. Exploring the characteristics of *Salix alba.*'s salt tolerance would provide a theoretical reference for research on its salt stress adaptability mechanism and its use in coastal saline-alkali areas.

2 Materials and Methods

2.1 Test materials and test design

The test materials were collected from the germplasm resource nursery of Golden Beach Forest Farm in Huai'an County, Hebei Province. The branches of the *Salix alba.* were basically the same in terms of growth, and those that were robust and free of diseases and insect pests were selected after the leaves fell in December. The middle two-thirds of the selected branches were cut into 20 cm-long cuttings. The uppermost bud was 0.5-1 cm from the top of the cuttings. The upper cut was a flat cut, and the lower cut was an oblique cut. The experiment was performed in the Artificial Climate Room of Hebei Agricultural University, Baoding City, Hebei Province on December 23, 2019. The temperature of the climate room was set to 28 ℃/25 ℃ (light/dark); the LED cold light source maintained the light intensity at 1000 $\mu mol \cdot m^{-2} \cdot s^{-1}$; the photoperiod was 14 h/10 h (light/dark); and the humidity was 60%.

The test material was placed in a 55 cm×38 cm×15 cm (length ×width ×height) plastic box for hydroponic culture(Fig 1). The experiment consisted of 5 treatments, and each treatment was repeated 3 times. We set the concentration of Nacl and the composition of culture medium by referring to existing studies[23,24,25]. A 1/2 dilution of Hoagland's complete nutrient solution was used as the base to prepare hydroponic solutions with NaCl concentrations of 0.1%, 0.2%, 0.3%, and 0.4%, 171mmol/L、342mmol/L、513mmol/L、684mmol/L, and 1/2 Hoagland's complete nutrient solution (PH=7.2) was used as a control (CK). 1/2 Hoagland's complete nutrient solution includes: $Ca(NO_3)_2 \cdot 4H_2O$ 472.5mg/L、K_2SO_4 303.5mg/L、$NH_4H_2PO_4$ 57.5mg/L、$MgSO_4$ 246.5mg/L、$NaFeC_{10}H_{12}N_2O_8 \cdot 3H_2O$ 30mg/L、$FeSO_4$ 15mg/L、H_3BO_3 2.86mg/L、$Na_2B_4O_7 \cdot 10H_2O$ 4.5mg/L、$MnSO_4$ 2.13mg/L, $CuSO_4$ 0.05mg/L、$ZnSO_4$ 0.22mg/L、$H_8MoN_2O_4$ 0.02mg/L. There were 25 cuttings in each treatment, and they were soaked directly in the solution; the height of the solution was approximately more than half of the height of the

cuttings. The nutrient solution was changed every 5 days during the growth process. Before the nutrient solution was changed, the cuttings were removed and the roots were rinsed with water to wash away the last residual salt and prevent excessive salt accumulation. The contents of Na^{+}, K^{+} and Ca^{2+} ions and the photosynthetic parameters and chlorophyll fluorescence kinetic curve parameters in the roots and leaves were determined following 20 days of treatment.

2.2 Measured items and methods

2.2.1 Measurement of root growth parameters

During the growth of *Salix alba.*, the number of root sprouting days and the rooting rate of all the cuttings were counted, and the rooting index was calculated according to the number of root sprouting days (rooting index = ∑Gt/Dt, where Dt, the day of the rooting test; Gt, the number of rooting branches on the day, and the rooting index is the number of rooting branches on the day/sum of days). After 20 days of salt stress treatment, 5 uniformly growing cuttings were selected to measure the average root number and average root length.

2.2.2 Determination of ion contents in the roots and leaves, calculation of the ion selective absorption and transport ratio

The measurement method for tracking the ion content was slightly modified relative to the method by Yang Sheng et al. [26] and Yu Bingjun et al. [27]. The sample was first baked at 105°C for 30 min and then dried at 70-80°C to a constant weight. After the sample was ground and passed through a sieve (the aperture was 0.425 mm), the fixed mass was weighed. Thirty mL of deionized water was added to the sample, which was then shaken well and placed in a boiling water bath for 2 h. After cooling, the sample was filtered and diluted to 50 mL. The Na^{+}, K^{+} and Ca^{2+} contents were determined by atomic absorption method (Atomic absorption spectrometer: ZEEnit700-700P; analytikjena computer in Germany). The methods of Zheng Qingsong et al. [28] and Yang Xiaoying et al. [29] were used to calculate the selective absorption and transport coefficients of ions X (K^{+} and Ca^{2+}) by the roots and leaves according to the following formula. Ion absorption coefficient $SA_{x, Na}$ =root ([X]/[Na^{+}])/medium ([X]/[Na^{+}]); ion transport coefficient $ST_{x, Na}$ = leaf ([X]/[Na^{+}])/Root ([X]/[Na^{+}]). In the formula, the K^{+} content in the medium (culture broth) was 272 mg/L, and the Ca^{2+} content was 230 mg/L.

2.2.3 Determination of photosynthetic parameters in the leaves

Following 20 days of salt stress treatment, the photosynthetic gas exchange parameters of the *Salix alba*. leaves were measured. Five uniformly growing cuttings were selected for each treatment group in the test. After the cuttings were left under normal illumination in the climate room for 3 hours, we selected the 3rd to 5th leaves from the top to bottom with the same position, size, and light-receiving direction and with fully expanded functional leaves. Using a Li-6800 portable photosynthesis meter (LI-COR, USA), the P_n, E, g_s and C_i can be determined. The measurement conditions were as follows: the PAR was 1000 $\mu mol \cdot m^{-2} \cdot s^{-1}$, the CO_2 concentration in the fixed system was 400 $\mu mol \cdot mol^{-1}$, and the relative humidity was 60%.

2.2.4 Rapid determination of the chlorophyll fluorescence induction kinetic curve

After 20 days of salt stress treatment, 5 *Salix alba*. cuttings with average growth were selected from each treatment for measurement. Before the measurement, the leaves were dark-adapted for 15 minutes, and then the rapid chlorophyll fluorescence induction kinetic curve and related parameters were measured using a Pocket PEA plant efficiency analyzer (Pocket PEA, Hansatech, UK). The resulting O-K-J-I-P curve was used for rapid chlorophyll fluorescence induction curve data analysis (JIP-test) and calculation [30,31].

2.3 data processing

One-way ANOVA and the LSD method were used to test the significance of the differences (α =0. 05).

3 Results

3.1 Effects of salt stress on *Salix albicans* root growth

The test results show that although plants under salt stress can reach a 100% rooting rate between treatments, the average root number, average root length and rooting index are quite different among the treatments, and the overall trend is basically the same. The trend is that low-salt stress stimulates root germination and elongation, high-salt stress inhibits root growth, and the intensity of the inhibition is positively correlated with the salt concentration.

Table Figs 2 and 3 show that when the NaCl concentration was 171mM/L, the average root number and average root length were significantly increased compared with those of the control. This result may be a stimulating effect of low-salt stress on root growth and then appear again as the stress intensifies, with a gradual downward trend. When

the NaCl concentration was 513mM/L, the root number and length were significantly lower than those of their respective controls by 48.7% and 39.9%, and the root growth was significantly inhibited at that time. Compared with the control, the rooting index did not change significantly when the NaCl concentration was 171mM/L, but with the increase in stress, the number of days for root germination was delayed, and the rooting index decreased significantly. When the NaCl concentrations were 342mM/L, 513mM/L and 684mM/L, the rooting indexes were significantly lower than that of the control (9.6%, 18.1% and 27.7%).

3.2 Effects of salt stress on ion content, absorption and transport in the roots and leaves of *Salix alba*

The ion content measurements (Table Fig 4) showed that under different concentrations of NaCl, the Na^+ contents in the roots and leaves of *Salix alba*. were significantly higher than that in the control group, and the range of Na^+ change was positively correlated with the stress concentration. The comparison of Na^+ contents in the roots and leaves shows that the Na^+ content of the roots is much higher than that in the leaves. Under 684mM/L NaCl stress, the Na^+ content in the roots could reach twice that in the leaves. With increasing stress concentration, the K^+ content in the leaves first increased and then decreased, reaching a peak at a concentration of 171mM/L NaCl, which was a significant increase of 14.0% compared to the control group. However, after the NaCl concentration was greater than 342mM/L, the concentration was significantly lower than that of the control. As the stress concentration increased, the K^+ contents in the roots of each treatment group showed a gradual decrease, which were all significantly lower than that of the control. The Ca^{2+} content in the leaves of *Salix alba*. increased first and then decreased with increasing salt concentration. At 342mM/L NaCl, compared with the control group, the concentration significantly increased by 13.6% and then showed a significant downward trend. The Ca^{2+} content in the roots decreased continuously with increasing stress, and when the NaCl concentration was 684mM/L, the Ca^{2+} content dropped to 35.6% of the control.

Table Figs 5 and 6 shows that both the Na^+/K^+ and Na^+/Ca^{2+} in the roots and leaves increased significantly with increasing NaCl stress concentration. This finding shows that as the stress intensifies, the relative absorption of Na^+ by *Salix alba.* increases greatly, but the absorption of K^+ and Ca^{2+} decreases. The Na^+/K^+ and Na^+/Ca^{2+} contents of all the treatments gradually decreased from root to leaf, and the rising Na^+/K^+ (F=1263.766, df=4， Sig.<0.001) and

Na^+/Ca^{2+} (F=10485.256, df=4，Sig.<0.001) in the roots were significantly higher than those in leaves (F=1235.223, df=4，Sig.<0.001; F=2335.783, df=4，Sig.<0.001), suggesting that *Salix* willow could reduce the salt stress damage to young tissues by regulating ion transport.

As shown in Table Fig 7, with increasing NaCl stress, the $SA_{k, Na}$, $ST_{k, Na}$, $SA_{Ca, Na,}$ and $ST_{Ca, Na}$ all showed a trend of first increasing and then decreasing. When the NaCl concentration was less than or equal to 342mM/L, the selective absorption capacity of the roots for K^+ (F=998.922, df=4，Sig.<0.001)and Ca2+ (F=1018.689, df=4，Sig.<0.001) and the selective transport capacity of the leaves for K^+ (F=168.047, df=4，Sig.<0.001) and Ca^{2+} (F=29.925, df=4，Sig.<0.001) were enhanced and reached a significant level. The selective absorption capacity of roots for K^+ is greater than that of Ca^{2+}, but the selective transport capacity of the leaves to Ca^{2+} is greater than that of K^+. These results indicated that *Salix* willow could adjust the upward transport capacity of K^+ and Ca^{2+} via the selective absorption and accumulation of mineral ions to compensate for the change in concentration under salt stress, to prevent the impacts of nutrient deficiency and ion toxicity on the shoot growth.

3.3 Effects of salt stress on photosynthetic parameters in *Salix alba*.Leaves

Table Figs 8 and 9 shows that the photosynthetic parameters of *Salix alba*. leaves were affected to different degrees under different salt concentrations. When the NaCl concentration was 171mM/L, the P_n of the leaves increased, but there was no significant difference from the control. Later, as the salt stress intensified, the photosynthetic carbon assimilation ability of *Salix alba*. leaves was significantly inhibited (F=95.66, df=4，Sig.<0.001); when the NaCl concentration was greater than 171mM/L, both the E (F=100.091, df=4，Sig.<0.001)and g_s (F=69.346, df=4，Sig.<0.001) were significantly lower than the control and became stronger; but at a low salt concentration (171mM/L NaCl), there is no significant difference from the control. With the increased salt concentration, the leaf C_i showed a trend of first decreasing and then increasing, reaching the lowest when the salt concentration was 342mM/L, which was significantly lower than the control by 10.4%, and then it gradually increased. The C_i of leaves (F=20.50, df=4，Sig.<0.001) under 513mM/L and 684mM/L NaCl treatments were not

significantly different from that of the control, but they were significantly higher than the lowest value by 12.4% and 14.6%, respectively.

3.4 Effect of salt stress on the rapid chlorophyll fluorescence induction kinetic curve (OJIP) of *Salix alba.*Leaves

The OJIP curve can provide a great deal of photochemical information about PS II and accurately reflect the state of the plant photosynthetic apparatus and the electron redox state of the PS II donor side, acceptor side and PS II reaction center in the photoreaction [32], thus representing the effects of external stress on the plant photosynthesis ability and even the degree of damage to the photosynthetic organs. Fig 10 shows that with of the increasing NaCl concentration, the OJIP curve of *Salix alba*. leaves changes to different degrees. Compared with the control group, under the 171mM/L NaCl treatment, the fluorescence value of JIP does not change significantly; when the NaCl concentration reaches 342mM/L and higher, the fluorescence values of I and P drop significantly and there is an obvious inflection point K (approximately 300 μs), and the OJIP curve changes to the O-K-J-I-P curve. The K-phase fluorescence value under high salt treatment is higher than that under low salt treatment, and the maximum fluorescence can be reached faster, which indicates that the higher the salt treatment concentration is, the greater the damage to the leaves of *Salix alba*.

3.5 Effects of salt stress on quantum yield and energy distribution ratio

Table Fig 11 shows that under different salt stresses, the energy absorbed, transformed, used for electron transfer, and dissipated by thermal radiation in the leaves of *Salix alba*. changes. Compared with the control group, with the increasing NaCl concentration, the maximum photochemical efficiency (φPo) of *Salix alba*. leaves after dark adaptation gradually decreased. Under the 342mM/L NaCl treatment, the φPo was significantly lower than that of the control. At that time, salt stress triggered photoinhibition, and the photosynthetic capacity of the leaves was reduced.

The excitons captured by the reaction center transfer electrons to the electron transport chain, and the ratio of excitons that exceed Q_A's other electron acceptors to promote Q_A reduction excitons (Ψo) and the light energy

absorbed by the reaction center are used for electron transfer. The quantum yields (φEo) all increased first and then decreased with the increasing salt stress. At 171mM/L NaCl, although the Ψo and φEo increased, they were not significantly different from the control. Later, as the stress intensified, both the Ψo and φEo were significantly lower than those of the control. When the NaCl concentration was 342mM/L, the Ψo and φEo were significantly lower than the 11.1% and 11.9% of the control group, respectively. Compared with the control group, salt stress increased the quantum ratio (φDo) of *Salix alba*. leaves for heat dissipation. When the NaCl concentration was 513mM/L, φDo was significantly higher than that of the control.

3.6 Influence of salt stress on the performance index and driving force

The performance index and driving force can accurately reflect the changes in the state of the photosynthetic apparatus of plants under stress. PI_{ABS} refers to the performance index based on the absorption of light energy, PI_{CSm} refers to the performance index based on the unit area, and DF_{CSm} refers to the driving force based on the unit area of the material. Table Figs 12 and 13 shows that as the NaCl stress concentration increases, PI_{ABS}, PI_{CSm} and DF_{CSm} all show a gradual decline. PI_{ABS} showed no significant difference from the control when the NaCl concentration was 171mM/L, and then with the increased salt concentrations, the difference became more significant (F=61.074, df=4，Sig.<0.001) , indicating that the *Salix alba.* leaves experienced photoinhibition, the PS II was damaged, and the measurement at the 684mM/L NaCl concentration was significantly lower than that of the control, by 60.2%. When the NaCl concentrations were 342mM/L, 513mM/L and 684mM/L, the PI_{CSm} values were significantly lower (F=202.821, df=4，Sig.<0.001) than that of the control by 20.1%, 43.9% and 66.4%; when the NaCl concentrations were 513mM/L and 684mM/L, the DF_{CSm} values were significantly lower (F=40.755, df=4，Sig.<0.001) than the control by 6.3% and 11.2%. Salt stress seriously affects the absorption of light energy by plants and leads to a decline in the basic driving force.

4 Conclusions and discussion

4.1 Influence of salt stress on the root growth status of *Salix alba*

As the primary organ responsible for plant material exchange, the root system and its growth status are closely related to the growth and development of the aboveground plant parts, whether the root system can function normally, and the plant's water and nutrient utilization efficiency [33]. Under salt stress, the root system is the first to feel the adversity stress signal, and it is also the most directly affected part [34]. Its ring-stripe inhibition is primarily manifested in the low levels of the root length, surface area and other parameters, and the root system grows slowly. A high-salt environment will cause plants to experience osmotic stress and ion toxicity, which will lead to changes in membrane permeability, which will in turn affect the absorption of water and nutrient elements by the roots, causing the plants to lose a large amount of water; the ions near the roots will be unbalanced, the physiological functions of the roots will eventually be lowered, and even the structure will be destroyed. Some of the aboveground leaves wilt, and photosynthetic production cannot be performed normally, which causes plant growth and metabolic disorders until the loss of physiological functions.

The change in root growth and the time of the root sprouting period can directly reflect the degree of damage to plants by salt stress and represent the strength of plant salt tolerance [35]. This study showed that the 171mM/L NaCl concentration significantly promoted the increase in the average number of roots and the elongation of the average root length of *Salix alba*. cuttings, and it can promote the rooting of the root system in advance, to a certain extent, which is consistent with Wang Shufeng et al. [36] and Ci Dun. The research results of Wei et al. [35] were basically the same. This growth response may be due to the decrease in water potential outside the roots under salt stress, which stimulates the growth of the roots instead of moderate osmotic stress to ensure the normal absorption of water and nutrients to meet the physiological and metabolic needs of the aboveground parts.

Some plants do have the phenomenon that low salt promotes the increase of some indicators, such as: promoting the germination of sorghum seeds[37], the roots of the seedlings of wolfberry[38] and rice[39], and the growth indicators of corn[40,41].Both Chorophy II in chrysanthemum [42] and proline content of cherry seedlings [43] are increased, while the net photosynthetic rate of wild chrysanthemum [44] and hazel trees increased [45]. The reason for the low salt concentration may be that the salt stress has a dual effect of stimulus and inhibition on plants. The strong and weak

relationship between stimulus and inhibition triggers changes in various plant indicators, resulting in the same low salt. It can promote growth, and it will be inhibited after high salt.This finding shows that *Salix alba*. has some ability to adjust and adapt to salt stress, and this adaptability is of great significance to the survival and continuation of the plant itself under adversity. However, as the salt stress intensifies, the ability of plants to coordinate their own growth is destroyed, root germination and elongation are significantly inhibited and become more intense, the root functions are destroyed, and the plants cannot maintain their normal growth and development.

4.2 Effects of salt stress on ion content, absorption and transport in *Salix alba*

Ions play an important role in the normal growth of plants, but salt stress can destroy the dynamic balance of ions in plants [46], hinder the absorption of nutrients, and cause plant metabolism disorders. The change in the distribution of ions reflects the degree of damage to plant cells by the external adverse environment. Additionally, plants can maintain balanced nutrition by improving the absorption and transport of ions, which also represents the level of plant resistance to stress. When measuring the ion contents of plant roots and leaves, it is helpful to reveal the salt tolerance or salt damage mechanism of plants.

In this study, when the salt concentration was low, the growth of *Salix alba*. was basically normal, the symptoms of salt damage were not significant, and the damage was obvious under severe stress. Na^+ accumulates significantly in the roots and leaves of *Salix alba*. under salt stress, but the Na^+ content in different organs is significantly different, and it is primarily concentrated in the roots. This result shows that the willow root system has a compensation mechanism that can reduce the transportation of salt to aboveground parts by enriching Na^+ in the root, thereby effectively reducing or delaying the occurrence of salt damage in the aboveground parts. This conclusion is consistent with the study by Hao Han et al. [47]. When the salt stress is too high, this balance is broken, and growth is blocked.

As an important inorganic solute, K^+ is essential for reducing the cell osmotic potential and maintaining the water balance. Generally, plants have an antagonistic effect on the absorption of Na^+ and K^+ [48], and the competition between the two usually leads to a decrease in the K^+ content. The loss of K^+ will cause changes in the physical structure of the stomata, frustrating photosynthesis [49]. In addition, K^+ participates in the metabolism of various enzymes in plants [50]. As salt stress increases, an excessive loss of K^+ will lead to K^+ dependent enzymes in *Salix*

alba. The enzyme activity decreases, which affects the metabolic reactions in which it participates. Therefore, if plants are to grow in a salty environment, the selective absorption of K^+ by the root system and the transportation of K^+ to the ground are particularly important. This study showed that the K^+ content in the roots of *Salix alba*. significantly decreased with increasing stress, but the K^+ in the leaves could be maintained at a high level at a 342mM/L NaCl concentration and below and even increased significantly when the NaCl concentration was 171mM/L, according to Zhou Qi et al. [51] A study on *Carpinus chinensis* also confirmed this result. At this time, the value and increase of Na^+/K^+ in the roots of the *Salix alba*. were greater than that of the leaves, and the $SA_{k, Na}$ and $ST_{k, Na}$ all increased significantly. Studies have shown that under salt stress, the Na^+/K^+ value can represent the degree of salt damage to the plant, and the lower Na^+/K^+ value of the leaves can help the plant better maintain its growth and photosynthetic function [52], and the $SA_{k, Na}$ and $ST_{k, Na}$ indicates that the plants can better tolerate salt stress [53]. This result shows that at that time, *Salix alba*. could maintain a relatively stable leaf K^+ content and the normal progress of photosynthesis by restricting the transportation of Na^+ from the root to the leaves, increasing the selective absorption of K^+ through the plant roots and the ability to transport K^+ to the ground. The accumulation of Na^+ causes damage to plants, which may be an important mechanism by which *Salix alba*. copes with salt stress. Later, with the increase in salt stress, the K^+ in the roots and leaves clearly flowed out. A high concentration of Na^+ will replace the Ca^{2+} bound to the membrane system, which will damage the integrity of the membrane structure and membrane function, thereby destroying the ion balance in the plant body and causing a large amount of organic solute extravasation [54]. The establishment of Ca^{2+} homeostasis in the cytoplasm is a key condition for salt adaptation [55]. This experiment showed that as the salt stress intensified, the Ca^{2+} content in the *Salix alba*. roots continued to decrease, but it could accumulate in the leaves when the NaCl concentration was ≤342mM/L. The results of Jia Yin et al. [56] were similar; the Na^+/Ca^{2+} value of white *Salix* roots was higher than that of the leaves, and the $Sa_{Ca, Na}$ and $ST_{ca, Na}$ were all significantly increased. This result may be due to the large influx of Na^+ into the root system under

salt stress, activating Ca^{2+} signal transduction, triggering the sodium elimination system to reduce the damage of Na^+, and enhancing the selective absorption of Ca^{2+} in leaves, thereby enhancing the selective transport of Ca^{2+} from root to shoot to maintain the low cell osmotic potential and the stability of the cell membrane. In addition, studies have shown that the increase in intracellular Ca^{2+} contents under salt stress can inhibit the outflow of K^+, thereby alleviating the damage of salt stress to plants [57]. Therefore, the upward transportation of Ca^{2+} in the roots of *Salix alba*. may be an important mechanism for it to maintain the balance of K^+ and Na^+ in the aerial part, establish ion homeostasis in the aerial part, and adapt to salt stress. However, due to the limited ability of the roots of *Salix alba*. to absorb Ca^{2+}, under high salt stress, the absorption of the roots will not be able to offset the loss of nutrient elements caused by ion poisoning.

4.3 Effects of salt stress on photosynthetic parameters of *Salix alba*

Photosynthesis is a key metabolic process that provides material energy for plants. High salt stress will comprehensively affect the photosynthesis of plants through osmotic stress, ion toxicity, and feedback inhibition caused by the accumulation of photosynthetic products [58]. These effects will cause the destruction of the membrane structure and the imbalance of ions in tissue cells, affecting the absorption of light energy by plants and the process of carbon assimilation [59]. This change inhibits the formation of leaf primordia and reduces the photosynthetic area and carbon assimilation of individual plants, resulting in physiological metabolic disorders and the accumulation of toxic substances. In fact, the energy supply related to photosynthesis, carbohydrate metabolism, and the TCA cycle are all inhibited by salt stress [60].

Because stomata are directly connected to the external environment, their coordinated response under stress determines whether the photosynthetic capacity of the plant is normal [61]. In this experiment, the P_n, E, and g_s did not change significantly when the NaCl concentration was 171mM/L. As the salt concentration further increased, each index decreased significantly, which is basically consistent with the results of previous studies [62,63]. When the NaCl concentration was less than 342mM/L, the C_i of the *Salix alba*. leaves decreased with decreasing g_s. Thus, the diffusion resistance of CO_2 in the leaves increases, and the carbon sequestration ability weakens. The stoma factor is

the dominant factor restricting the decline in *Salix alba*. leaf photosynthesis. Later, as the degree of salt stress further intensified, the C_i increased with the decreasing g_s, and the photosynthetic system activity of the mesophyll cells decreased, resulting in a decrease in the assimilation capacity, which is a typical non-stomatal limiting factor. Previous studies have shown that under adverse stress, stomatal restriction and non-stomatal restriction and the interaction of the two will reduce the photosynthetic rate of plants; under mild stress, stomatal restriction is dominant; and under severe stress, stomatal restriction leads to non-stomatal restriction [64,65]. Our experiment also supports this view.

4.4 Effects of salt stress on chlorophyll fluorescence kinetics of *Salix alba*

The OJIP curve contains a great deal of information about the original photochemical reaction of the PSII reaction center [66]. When environmental conditions change, chlorophyll fluorescence can directly or indirectly affect the photosystem performance of plants [67]. The changes in the PSII can reflect the impact of changes in the stress environment on the photosynthetic capacity of plants and the adaptation mechanism of photosynthetic machinery to environmental changes. High salt stress can inhibit or destroy parts of the functions of PS II, hinder the original photochemical reaction and electron transfer process of PS II, and reduce the photosynthetic capacity of *Salix alba*. leaves. This consequence may be the result of the accumulation of Na^+. The typical fast fluorescence kinetics curve generally has O, J, I, and P phases during the rising phase of fluorescence [68]. This study shows that when the concentration of NaCl is ≥342mM/L, the OJIP curve of *Salix alba*. will be deformed to OKJIP, the fluorescence values of points I and P will decrease significantly, and obvious inflection point K will appear. The occurrence of the K point is caused by damage to the PSII donor side oxygen release complex (OEC) due to the inhibition of the water lysis system and the receptor-side part before Q_A, and the relatively variable fluorescence of the K point can represent the degree of OEC damage [69,70]. In addition, the high salt treatment greatly shortened the time required to reach the P point (the maximum fluorescence value). This result indicates that the higher the degree of salt stress, the greater the damage to the stability of the PS II reaction center and the OEC on the PS II donor side of *Salix alba*. leaves, the weaker the ability to provide electrons downstream and the stronger the reduction of the PS II acceptor side is hindered.

The φPo, Ψo, φEo, φDo reflect the energy distribution ratio of plants. In this study, when the NaCl concentration was 171mM/L, there was no significant difference among the indicators. As the stress intensified, the φPo, Ψo and φEo decreased significantly while the φDo increased significantly, which is different from the results of Huang

Qinqin et al. [71]. This finding shows that *Salix alba*. adjusted the energy distribution ratio of the PSII reaction center under different degrees of stress. This adjustment occurs to increase the quantum ratio used for heat dissipation and reduce the proportion of energy in photochemical reactions, which is an adaptive regulation mechanism of *Salix alba*. under salt stress. The decrease in the φPo, Ψo and φEo indicates that the photosynthetic machinery is clearly damaged, the ability to reduce the Q_B and P_Q on the PSII receptor side is diminished, and the electron transfer process is inhibited. Plants are prone to occur or aggravate photoinhibition in adverse environments [69]. In this study, when the concentration of NaCl was greater than 171mM/L, the PI_{ABS}, PI_{CSm} and DF_{CSm} all showed a significant downward trend. This trend shows that *Salix alba*. leaves exhibit photoinhibition, the PSII reaction center is reversibly inactivated or irreversibly degraded, the conversion efficiency of light energy is reduced, and the function of the photosynthetic apparatus is impaired, which restricts the normal progress of photosynthesis.

In this study, 171mM/L NaCl stress had no significant effect on the growth status of the Salix alba. root system, ion distribution or photosynthetic fluorescence characteristics and even increased these parameters to a certain extent. As the salt treatment concentration gradually increased, the average root number, average root length, and rooting index decreased significantly; Na^+ accumulated in the root system, K^+ and Ca^{2+} were significantly lost; the photosynthetic rate decreased significantly, the PS II reaction center was partially inactivated, and the donor side OEC and the electron acceptor on the acceptor side were damaged. *Salix alba* can respond to salt stress by intercepting Na^+ in the root system, improving the selective absorption of K^+ and Ca^{2+} and the ground transportation capacity, and increasing the quantum ratio used for heat dissipation, indicating that *Salix* willow has some tolerance to salt stress environments.

Supporting Information

S1 Table. Effects of salt stress on the contents of three ions in roots and leaves of *Salix alba L*

Acknowledgments

We express sincere gratitude to all the authors involved in this study.

Author Contributions

Conceptualization: Xin Ran, Xiao Wang

Data curation: Xin Ran, Xiao Wang, Xiaokuan Gao, Haiyong Liang, Bingxiang Liu, Xiaoxi Huang

Formal analysis: Xiao Wang, Xin Ran, Bingxiang Liu,

Funding acquisition: Haiyong Liang, Bingxiang Liu

Methodology: , Xiaokuan Gao, Bingxiang Liu

Resources: Bingxiang Liu, Xiao Wang

Writing-original draft: Xiao Wang, Bingxiang Liu

Writing-review&editing: Xiaokuan Gao, Haiyong Liang, Bingxiang Liu

References

1. ZHANG J L, FLOWERS T J, WANG S M (2010) Mechanisms of sodium uptake by roots of higher plants. Plant and Soil 326(1-2): 45-60.
2. MA W Y (2004) Progress in research of plant tolerance to Saline Stress. Agriculture and Technology 24(04): 95-99. [in Chinese]
3. ZHU J F, CUI Z R, WU C H, et al (2018) Research Advances and Prospect of Saline and Alkali Land Greening in China. World Forestry Research 31(04): 70-75. [in Chinese]
4. YANG Q S, LIU D, GAO W D, et al (2016) Research Progress on Stress Resistance of Willow. Forestry Science and Technology Newsletter (07): 24-27. [in Chinese]
5. WANG Q Z, LIU Q, GAO Y N, et al (2017) Review on the mechanisms of the response to salinity-alkalinity stress in plants. Acta Ecologica Sinica 37(16): 5565-5577. [in Chinese]
6. LI Z Y (2017) Physiological responses of Salix psammophila seedlings to complex saline-alkali stress. Beijing: Chinese Academy of Forestry sciences. [in Chinese]
7. LIU Y M, CHENG C, JIANG L, et al (2019) Comparisons on growth, salt ion distribution, and relative expression of SOS1 gene in three species of Tamarix Linn. under NaCl stress. Journal of Plant Resources and

Environment 28(01): 1-9. [in Chinese]

8. ZHANG X, HE K N, SHI C Q, et al (2017) Effects of salt stress on growth and physiological characteristics of Tamarix chinensis and Nitraria tangutorum seedlings. Journal of Northwest A & F University (Natural Science Edition) 45(01): 105-111. [in Chinese]

9. LI Z Y, CONG R C, YANG Q S, et al (2017) Effects of saline-alkali stress on growth and osmotic adjustment substances in willow seedlings. Acta Ecologica Sinica 37(24): 8511-8517. [in Chinese]

10. ZHANG J L, LI H R, GUO S Y, et al (2015) Research advances in higher plant adaptation to salt stress. Acta Prataculturae Sinica 24(12): 220-236. [in Chinese]

11. YANG S, LIU Z X, ZHANG H X, et al (2013) Comprehensive evaluation of salt tolerance and screening identification indexes for three tree species. Scientia Silvae Sinicae 49(1): 91-98. [in Chinese]

12. RAUT R, SHARMA S, BAJRACHARYA R M. (2012) Biotic response to acidification of lakes: a review. Kathmandu University Journal of Science, Engineering and Technology 8(1): 171-184.

13. KALAJI H M, JAJOO A, OUKARROUM A, et al (2016) Chlorophyll a fluorescence as a tool to monitor physiological status of plants under abiotic stress conditions. Acta Physiologiae Plantarum 38(4): 1-11.

14. Kolozsvári Ildikó,Kun Ágnes,Jancsó Mihály, et al (2021) Utilization of Fish Farm Effluent for Irrigation Short Rotation Willow (Salix alba L.) under Lysimeter Conditions. Forests 12(4).

15. Barnes J, Anderson LA and Phillipson JD. (2007) Herbal Medicines.London. Chicago: Pharmaceutical Press :598-600 .

16. HUI Y H, WANG R C.(2004) Determination of salicin in the extract of Salix officinalis by RP-HPLC. Chinese Traditional and Herbal Medicines (05):48-49.[in Chinese]

17. KONG J H, WANG Y F, YANG X F, et al (2012) Extraction of salicin from populus euphratica leaves by alcohol-alkali method . Food science and technology 37(11):205-209.[in Chinese]

18. DUAN H, ZHAI K F, GAO G Z, et al (2012) Purification of salicin from the roots of maple leaf by macroporous adsorption resin. Food science 33(22):99-102.[in Chinese]

19. Mataruga Z , S Jarić, M Marković, et al (2020) Evaluation of Salix alba, Juglans regia and Populus nigra as biomonitors of PTEs in the riparian soils of the Sava River. Environmental Monitoring and Assessment 192(2):131.

20. Bajraktari Demush,Petrovska Biljana Bauer, Zeneli Lulzim, et al (2020) Soil chemical evaluation and power plant ash impact on chemical properties of Salix alba L. (Fam. Salicaceae): The impact of bioaccumulation. Toxicology Research and Application 4(4).

21. Regni Luca,Bartucca Maria Luce,Pannacci Euro, et al (2021) Phytodepuration of Nitrate Contaminated Water Using Four Different Tree Species.Plants (Basel, Switzerland) 10(3).

22. A. S. Quiñones Martorello,M. E. Fernάndez,M.G. Monterubbianesi, et al (2020) Effect of combined stress (salinity + hypoxia) and auxin rooting hormone addition on morphology and growth traits in six Salix spp. clones. New Forests: International Journal on the Biology, Biotechnology, and Management of Afforestation and Reforestation 51(1).

23. HU A S, ZHANG X D, GUO W J, et al(2021) Ion Absorption, transport and distribution of Malus petalis under salt Stress. Plant Physiology Journal 57(09):1829-1838. [in Chinese]

24. LI B B, OU YJ, WANG J Y, et al(2017) Effects of NaCl on growth, development and some physiological characteristics of salix matsulosa. Journal of tianjin normal university (natural science edition) 37(06):37-42. [in Chinese]

25. CHEN, T,White,James F, (2021) Exogenous spermidine enhances Epichloë endophyte-induced tolerance to NaCl stress in wild barley (Hordeum brevisubulatum). Plant and Soil(prepublish).

26. YANG S (2010) Study on selection and evaluation criteria of salinity-tolerance tree species in coastal region. Beijing: Chinese Academy of Forestry sciences. [in Chinese]

27. YU B J, LUO Q Y, CAO A Z, et al (2001) Comparison of salt tolerance and ion effect in cultivated and wild soybean. Journal of Plant Resources and Environment 10(01): 25-29. [in Chinese]

28. ZHENG Q S, WANG R L, LIU Y L (2001) Effects of Ca^{2+} on Absorption and Distribution of Ions in Salt-treated Cotton Seedlings. Plant Physiology Journal 27(4): 325-330. [in Chinese]

29. YANG X Y, ZHANG W H, WANG Q Y, et al (2003) Salt tolerance of wild soybeans in Jiangsu and its relation with ionic distribution and selective transportation. Chinese Journal of Applied Ecology 14(12): 2237-2240. [in Chinese]

30. STRASSER R J, SRIVASTAVA A, TSIMILLI-MICHAEL M (2000) The fluorescence transient as a tool to characterize and screen photosynthetic samples. In: Yunus M, Pathre U, Mohanty P (eds). Probing Photosynthesis: Mechanism, Regulationand Adaptation. London: Taylor and Francis Press, 445–483.

31. STRASSER B J, STRASSER R J (1995) Measuring fast fluorescence transients to address environmental questions: The JIP test. In: Mathis P ed. Photosynthesis: From Light to Biosphere. Dordrecht, the Netherlands: KAP Press 5: 977~980.

32. SUN J, JIA Y X, GUO S R, et al. (2010) Resistance of spinach plants to seawater stress is correlated with higher activity of xanthophyll cycle and better maintenance of the chlorophyll metabolism. Photosynthetica 48(4): 567-579.

33. YANG Z Q, QIU Y X, LIU Z X, et al (2016) Effects of soil water stress on root and overground growth of plant tomato. Acta Ecologica Sinica 36(3): 748-757. [in Chinese]

34. WANG S F, HU Y X, SUN H J, et al (2014) Effects of salt stress on growth and root development of two oak seedlings. Acta Ecologica Sinica 34(4): 1021-1029. [in Chinese]

35. CI D W, ZHANG Z M, DING H, et al (2015) Evaluation and selection indices of salinity tolerance in peanut seedling[J]. Acta Ecologica Sinica 35(03): 805-814. [in Chinese]

36. WANG S F, HU Y X, LI Z L, et al (2010) Effects of NaCl stress on growth and mineral ion uptake, transportation and distribution of Quercus virginiana. Acta Ecologica Sinica 30(17): 4609-4616. [in Chinese]

37. CHEN X F,ZHANG R D,XINNG Y F, et al (2021) The efficacy of different seed priming agents for promoting sorghum germination under salt stress. PloS one 16(1)

38. ZHU J J, MA H J, QIN H Y. et al (2021) Effects of potassium solubilizing bacteria and salt stress on root characteristics and physiological metabolism of Lycium barbarum seedlings. Agricultural Research in the Arid Areas 39(05):50-58+65.[in Chinese]

39. LIU S H, ZHU X S, YAN M, et al (2020) Effects of NaCl immersion on root growth characteristics of hybrid rice seedlings under salt stress. Journal of southwest university (natural science edition)42(08):59-65.[in Chinese]

40. Iqbal M, Ashraf M, Jamil A, et al (2006) Does seed priming induce changes in the levels of some endogenous plant hormones in hexaploid wheat plants under salt stress. Journal of Integrative Plant Biology 48(2): 181 – 189.

41. Tsegay BA, Yohannes G (2013) The role of seed priming in improving seedling growth of maize (Zea mays L.) under salt stress at field conditions. Agricultural Sciences 4(12): 666 – 672.

42. LI Z L (2020) Evaluation of salt tolerance of four chrysanthemum and its related genera. Liaoning Forestry Science and Technology (02):36-38. [in Chinese]

43. [2] SHU J, LIU S H, ZHANG A H, et al (2021) Effects of NaCl stress on photosynthetic characteristics, chlorophyll fluorescence and osmotic regulatory substances in Cherry seedlings. Journal of Shanxi Agricultural Sciences 49(07):834-838.[in Chinese]

44. LIU X W, XIA B, CHEN B, et al (2021) Effects of salt Stress on photosynthetic physiology of Chrysanthemum and Chrysanthemum shennong and their F_1 hybrids. Journal of Northeast Forestry University 49(05):32-39.[in Chinese]

45. LUO D, SHI Y J, SONG F H, et al (2019) Effects of salt stress on growth, photosynthetic and fluorescence characteristics and root architecture of heterosis hazel seedlings. Chinese journal of applied ecology 30(10):3376-3384.[in Chinese]

46. RUIZ K B, BIONDI S, MARTINEZ E A, et al (2016) Quinoa – a model crop for understanding salt-tolerance mechanisms in halophytes. Plant Biosystems 150(2): 357-371.

47. HAO H, CAO L, CHEN W N, et al (2020) Effects of salt stress on the ion balance and physiological-biochemical characteristics of Quercus dentata seedlings. Acta Ecologica Sinica 40(19): 6897-6904. [in Chinese]

48. WANG N, QIAN W, LIU X, et al (2017) Relative contribution of Na^+/ K^+ homeostasis, photochemical efficiency and antioxidant defense system to different salt tolerance in cotton (Gossypium hirsutum L.) cultivars. Plant Physiology and Biochemistry 119: 121-131.

49. GUO P, WEI H X, ZHANG W J, et al (2016) Physiological responses of alfalfa to high-level salt stress: root ion flux and stomatal characteristics. International Journal of Agriculture and Biology 18(1): 125-133.

50. SINGH V, SINGH A P, BHADORIA J, et al (2018) Differential expression of salt-responsive genes to salinity stress in salt-tolerant and salt-sensitive rice (Oryza sativa L.) at seedling stage. Protoplasma 255(6): 1667-1681.

51. ZHOU Q, ZHU Z L (2015) Effects of NaCl stress on seedling growth and mineral ions uptake, distribution and transportation of two varieties of Carpinus L. Journal of Beijing Forestry University 37(12): 7-16. [in Chinese]

52. ZHU J K (2003) Regulation of ion homeostasis under salt stress. Current Opinion in Plant Biology 6(5): 441-445.

53. DONG F, CAO J, LI X T, et al (2016) Effects of various types of salt stress on ion absorption, accumulation and transportation in pea (Pisum sativum) seedlings. Acta Prataculturae Sinice, 25(11): 66-75. [in Chinese]

54. JIANAER.A, YANG C W, SHI D C, et al (2007) Physiological Response of an Alkali Resistant Halophyte Kochia sieversiana to Salt and Alkali Stresses. Acta Botanica Boreali-Occidentalia Sinica 27(1): 79-84. [in Chinese]

55. ZHAO K F (1997) halophyte. Chinese Bulletin of Botany (4): 2-13. [in Chinese]

56. JIA Y, XIANG Y F, WANG L L, et al (2020) Effects of salt stress on the growth and physiological characteristics of Primula forbesii. Acta Prataculturae Sinice 29(10): 119-128. [in Chinese]

57. HAN Y X, CHEN S L, ZHANG Y H, et al (2013) Exogenous hydrogen peroxide, nitric oxide and calcium mediate root ion fluxes in two non-secretor mangrove species subiected to NaCl stress. Tree Physiology 33(1): 81-95.

58. QI Q, MA S R, XU W D (2020) Research progress on the effects of salt stress on plant growth and the physiological mechanism of salt tolerance. Molecular plant breeding 18(8): 2741-2746. [in Chinese]

59. ZHANG R J, REN F, BAI Y B, et al (2012) Research progress on the influence of stress on PSII based on the dynamic analysis of fast chlorophyll fluorescence induction. Anhui Agricultural Science 40(7): 3858-3859, 3864. [in Chinese]

60. PANG Q Y, ZHANG A Q, ZANG W, et al (2016) Integrated proteomics and metabolomics for dissecting the

mechanism of global responses to salt and alkali stress in Suaeda corniculate. Plant and Soil 402(1/2): 379-394.

61. WANG Z Y, YANG Y, HUA J F, et al (2020) Effects of alkali treatment on photosynthetic characteristics and physiology of seedling leaves of Chinese fir 406. Journal of Plant Resources and Environment 29(04): 72-74. [in Chinese]

62. LI X F, NI Z M, WU Y Y, et al (2015) Effects of salt stress on photosynthetic characteristics and leaf cell structure of 'Yinhong' grape seedlings. Acta Ecologica Sinica 35(13): 4436-4444. [in Chinese]

63. YANG J Y, ZHENG W, TIAN Y, et al (2011) Effects of various mixed salt-alkaline stresses on growth，photosynthesis， and photosynthetic pigment concentrations of Medicago ruthenica seedlings. Photosynthetica 49(2): 275-284.

64. LI X X, LIU B X, GUO Z T, et al (2013) Changes of photosynthetic characteristics and fast chlorophyll fluorescence induced kinetic curve of rhizoma coptidis leaves under NaCl stress. Chinese Journal of Applied Ecology 24(9): 2479－2484. [in Chinese]

65. ZHANG H H, ZHANG X L, LI X, et al (2012) Effects of NaCl and Na_2CO_3 stress on growth and photosynthetic characteristics of mulberry seedlings. Chinese Journal of Applied Ecology 23(3): 625－631.

66. LI P M, GAO H Y (2005) Application of fast chlorophyll fluorescence induction kinetics analysis in photosynthesis. Journal of Plant Physiology and Molecular Biology 31(6): 559 -566. [in Chinese]

67. KONG F, WANG W C, HU H, et al (2019) Effects of NaCl Treatment on Photosynthetic and Fast Chlorophyll Fluorescence Characteristics in Herbaceous Peony. Molecular Plant Breeding 17(22): 7531-7537. [in Chinese]

68. HUANG Q X, ZHAO S, LIU C M, et al (2015) Effects of Shading Treatments on Chlorophyll Fluorescence Characteristics of Sabina vulgaris Seedlings Grown in Iron Tailings Media. Scientia Silvae Sinicae 51(6): 17-26. [in Chinese]

69. KONG F, LIU X Y, WANG G Z, et al (2016) Effect of fertilizer application on photosynthesis and fast chlorophyll fluorescence characteristics of walnut. Forestry scientific research 29(5): 764-769. [in Chinese]

70. LI H, LI H W, LV Y J, et al (2019) Salt priming protects photosynthetic electron transport against low-

temperature-induced damage in wheat. Sensors 20(1): 62.

71. HUANG Q Q, YANG Z Q, LI J S, et al (2019) Effect of Water and Nitrogen Coupling on Rapid Fluorescence Induction Kinetics Characteristics of Facility Grape Leaves. Chinese Journal of Agrometeorology 40(9): 557-573. [in Chinese]

Figur captions:

Fig.1 Experimental scenes of the effects of salt stress on *Salix alba L*

Fig. 2 Changes of root growth of *Salix alba L* under salt stress

Fig. 3 Effects of salt stress on root growth of *Salix alba* cuttings.

Fig. 4 Effects of salt stress on ion content in roots and leaves of *Salix alba L*

Fig. 5 Effects of salt stress on Na^+/ Ca^{2+} in roots and leaves of *Salix alba L*

Fig. 6 Effects of salt stress on Na^+/ K^+ in roots and leaves of *Salix alba L*

Fig. 7 Effects of salt stress on selective uptake and transportation of ion in roots and leaves of *Salix alba L*.

Fig. 8 Effects of salt stress on photosynthetic parameter (Pn and E)in leaves of *Salix alba L*

Fig. 9 Effects of salt stress on photosynthetic parameter (Ci and gs) in leaves of *Salix alba L*

Fig. 10 Effect of salt stress on the fast induction curves of chlorophyll a fluorescence（O-J-I-P curve）of *Salix alba L leave*

Fig. 11 changes of Chlorophyll a fluorescence parameters under salt stress of *Salix alba L*

Fig. 12 The changes of Performance index and driving force (PIABS and DFCSm)under different salt stress

Fig. 13 The changes of Performance index and driving force (PICSm)under different salt stress

Reply to all comments point-by-point (Manuscript Number: PONE-D-21-23173)

The authors' replies are in blue.

Editor's comments:

1. Please ensure that your manuscript meets PLOS ONE's style requirements, including those for file naming. The PLOS ONE style templates can be found at https://journals.plos.org/plosone/s/file?id=wjVg/PLOSOne_formatting_sample_main_body.pdf and https://journals.plos.org/plosone/s/file?id=ba62/PLOSOne_formatting_sample_title_authors_affiliations.pdf

[Reply] We have modified the manuscript according to the PLOS ONE's style requirements.

2. We note that you have stated that you will provide repository information for your data at acceptance. Should your manuscript be accepted for publication, we will hold it until you provide the relevant accession numbers or DOIs necessary to access your data. If you wish to make changes to your Data Availability statement, please describe these changes in your cover letter and we will update your Data Availability statement to reflect the information you provide.

[Reply] We will provide the relevant accession numbers or DOIs necessary to access our data.

3. PLOS requires an ORCID iD for the corresponding author in Editorial Manager on papers submitted after December 6th, 2016. Please ensure that you have an ORCID iD and that it is validated in Editorial Manager. To do this, go to 'Update my Information' (in the upper left-hand corner of the main menu), and click on the Fetch/Validate link next to the ORCID field. This will take you to the ORCID site and

allow you to create a new iD or authenticate a pre-existing iD in Editorial Manager. Please see the following video for instructions on linking an ORCID iD to your Editorial Manager account: https://www.youtube.com/watch?v=_xcclfuvtxQ

[Reply] We create a new iD:https://orcid.org/0000-0002-1699-7648

COMMENTS FOR THE AUTHOR:

Reviewer 1#

- The study is carefully concepted and methodological approach is satisfactory explained. The manuscript deals with interesting and important changes in response to salt stress on the photosynthetic physiology and mineral ion absorption and distribution in white willow (Salix alba L.). Results and discussion portions are well written. The authors also draw an accurate picture from the results.

[Reply] Thank you very much for your valuable feedback. We have made corrections based on the suggestions. Please see the manuscript.

- However, few parameters are missing. The authors should review and cite some more relevant references, and follow suggestion given in detail below.

[Reply] We have reviewed and cited more relevant references. Please see line 487-510 of the “Revised Manuscript with Track Changes”.

- Also needs a proper revision of English language. Many sentences are very confusing. English language and writing style needs to be improved sufficiently.

[Reply] We have modified the English language appropriately. Thank you very much for your valuable suggestions. We will continue our efforts to improve our English in the future.

- Do not repeat words of the title in the Keywords.

[Reply] We have rewrote Keywords. Please see line 41-42 of the “Revised Manuscript with Track Changes”.

- Please for the first use Salix alba L., then please follow the only Salix alba in the manuscript.

[Reply] We have rewrote the name throughout the manuscript. Please look at the example from line 15+18 of the “Revised Manuscript with Track Changes”.

- Add economic and other importance of plant in introduction to make it more valuable.

[Reply] We have added economic and other importance of plant in introduction. See Line 64-69 of the “Revised Manuscript with Track Changes” .

- It is better to NaCl concentration in mM instead of %.

[Reply] We've changed % to mM throughout the manuscript. Please see line 91 of the “Revised Manuscript with Track Changes”.

- Line: 25- 27. Please site the reference for hydroponics.

[Reply] We've cited the reference for hydroponics. Please see Line 89 +92-95+454-460 of the “Revised Manuscript with Track Changes” .

- Objective of the study needs to be refined.

[Reply] We have refined our research objectives. Please see Line 16-17 of the “Revised Manuscript with Track Changes”.

- Line: 98. Which instrument (its name model, company) used for the determination of ions?

[Reply] We have added information about this instrument. We used the atomic absorption spectrometer of Analytikjena in Germany for atomic absorption determination.Please see line 116-117 of the “Revised Manuscript with Track Changes”.

- In my opinion, chlorophyll content (a, b, and total) and carotenoids needs to be measured. It have direct link with salt stress and adaptability. NaCl and RWC have direct link when talking about stress and adaptability. So, I will suggest to study RWC (relative water content) under salt stress.

[Reply] This study mainly focuses on the effects of salt ions in plants on photosynthetic performance, without considering chlorophy II and water. Thank you for your valuable suggestions, which will be added and improved in our future studies.

- References need to be revised. Many articles on salinity response are published in high impact factor journals. So try to cite them, so everyone can access the references as well. Also, try to cite the latest articles.

[Reply] We have deleted some references. We have added some influential and recent articles. Please see line 487-510 of the “Revised Manuscript with Track Changes”.

COMMENTS FOR THE AUTHOR:

Reviewer 2#

- However, this mechanism seems to be general in many plants, and also, as lack of data, the conclusions can not be rich and for further discussion.

[Reply] Thank you very much for your suggestions. We have revised the manuscript. Most of the researches on White Willow mainly focus on the medicinal value of substances such as salicin contained in the bark, or the value of studying the enrichment of heavy metals in white willow, which is mainly used to purify water resources and realize agricultural irrigation and fishery breeding. Habitat stress is mainly drought and flooding, but there are few literatures on salt stress, most of which focus on the responses of physiological indexes and photosynthetic indexes of plants to ion

absorption and transport under salt stress. This is also one of the reasons for our research. We hope to use white willow as experimental material to observe the various effects of salt stress on plants from this perspective.

COMMENTS FOR THE AUTHOR:

Reviewer 3#

The manuscript titled "Effects of salt stress on the photosynthetic physiology and mineral ion absorption and distribution in white willow (Salix alba L.)" one of classic example of tree species response to salt stress. The manuscript is well organized and language easy to follow although there were few grammatical and Syntex errors besides SI units.

[Reply] Thank you very much for your suggestions. We have corrected grammatical and syntactic errors .Please see the manuscript.

-1. Adapting a hydroponic system to research salt stress may be the most recent best strategy, but there is no way of knowing how the experimental setting was done. Authors may include a photo of the same in order to make any relevant comments.

[Reply] We have added a picture to the manuscript. Please see the Figure 1.

-2.Similarly for rooting length, kindly provide some photo to understand plant response.

[Reply] We have added a photo. Please see the figure 2.

-3. Throughout the manuscript I can see that salt stress has a significant impact on above-ground biomass, such as leaf area index, shoot length, and so on, but the fact that it was not included in this study is a significant disadvantage. In the meantime, various physiological characteristics have been subjected leaves.

[Reply] This study mainly focuses on the effects of salt ions in plants on photosynthetic performance, without considering the aspects of leaves and stems. Thank you for your valuable suggestions, which will be added and improved in future research.

-4. When it comes to hydroponic systems, CK medium may be the best option, however, the reaction of Salix alba seedlings is substantially higher than 0.1% NaCl, but no justification from authors how or why although the salt concentration less than 0.1% NaCl.

[Reply] we have made an explanation, please see line 263-269 of the “Revised Manuscript with Track Changes”. Thank you very much for your valuable suggestions.

-5. A statistical analysis was performed, however it was not up to scientific merit. For example, the manuscript frequently mentions significance of attributes, but there are no ANOVA tables (except LSD rank), at least as a supplemental to support. I request the authors to include F=xxx, df=xxx, and sig.=>0.001 wherever statistical significance was indicated.

[Reply] We added some these information throughout the manuscript. Please see the example form line 173-175+179+181 of the “Revised Manuscript with Track Changes”.

6. Throughout the article, there are discussions about the trends of the morphological and physiological response of plant to the treatments, but the presented table does not provide viewpoints. Authors profusely mentioned the response trends widely without statistical analysis about trends which containing the scatter plot and best fit regression models (r2 and P). I advise authors to use graphs instead of tables, and I've included a sample presentation for ready reference.

[Reply] Thank you very much for your valuable suggestions. We have changed some tables into charts. Please see figure 7.
