# Supplementary material for: Effects of salt stress on the photosynthetic physiology and mineral ion absorption and distribution in white willow (Salix alba L.)
Source: PLoS One. 2021 Nov 18;16(11):e0260086. doi: 10.1371/journal.pone.0260086 (PMC8601552; doi:10.1371/journal.pone.0260086)
Supplement: Attachment — Submitted filename: PlosOne-D-21-23173_Review.docx [file pone.0260086.s002.docx]

The manuscript tittled “Effects of salt stress on the photosynthetic physiology and mineral ion absorption and distribution in white willow (*Salix alba* L.)” one of classic example of tree species response to salt stress. The manuscript is well organized and language easy to follow although there were few grammatical and Syntex errors besides SI units. The manuscript can be improved so many ways before making the technical comments and some of my major concerns are;

1. Adapting a hydroponic system to research salt stress may be the most recent best strategy, but there is no way of knowing how the experimental setting was done. Authors may include a photo of the same in order to make any relevant comments.
2. Similarly for rooting length, kindly provide some photo to understand plant response.
3. Throughout the manuscript I can see that salt stress has a significant impact on above-ground biomass, such as leaf area index, shoot length, and so on, but the fact that it was not included in this study is a significant disadvantage. In the meantime, various physiological characteristics have been subjected leaves.
4. When it comes to hydroponic systems, CK medium may be the best option, however, the reaction of *Salix alba* seedlings is substantially higher than 0.1% NaCl, but no justification from authors how or why although the salt concentration less than 0.1% NaCl.
5. A statistical analysis was performed, however it was not up to scientific merit. For example, the manuscript frequently mentions significance of attributes, but there are no ANOVA tables (except LSD rank), at least as a supplemental to support. I request the authors to include F=xxx, df=xxx, and sig.=>0.001 wherever statistical significance was indicated.
6. Throughout the article, there are discussions about the trends of the morphological and physiological response of plant to the treatments, but the presented table does not provide viewpoints. Authors profusely mentioned the response trends widely without statistical analysis about trends which containing the scatter plot and best fit regression models (r2 and P). I advise authors to use graphs instead of tables, and I've included a sample presentation for ready reference.

|  | SACa, Na |
| --- | --- |
| 0.1% | 0.166 |
| 0.2% | 0.235 |
| 0.3% | 0.163 |
| 0.4% | 0.12 |
