# Supplementary material for: Effects of salt stress on the photosynthetic physiology and mineral ion absorption and distribution in white willow (Salix alba L.)
Source: PLoS One. 2021 Nov 18;16(11):e0260086. doi: 10.1371/journal.pone.0260086 (PMC8601552; doi:10.1371/journal.pone.0260086)
Supplement: Attachment — Submitted filename: Response to Reviewers.docx [file pone.0260086.s003.docx]

**Reply to all comments point-by-point (Manuscript Number: PONE-D-21-23173)**

The authors’ replies are in blue.

**Editor’s comments:**

1. Please ensure that your manuscript meets PLOS ONE's style requirements, including those for file naming. The PLOS ONE style templates can be found at
[https://journals.plos.org/plosone/s/file?id=wjVg/PLOSOne_formatting_sample_main_body.pdf](https://journals.plos.org/plosone/s/file?id=wjVg/PLOSOne_formatting_sample_main_body.pdf) and [https://journals.plos.org/plosone/s/file?id=ba62/PLOSOne_formatting_sample_title_authors_affiliations.pdf](https://journals.plos.org/plosone/s/file?id=ba62/PLOSOne_formatting_sample_title_authors_affiliations.pdf)

[Reply] We have modified the manuscript according to the PLOS ONE's style requirements.

2. We note that you have stated that you will provide repository information for your data at acceptance. Should your manuscript be accepted for publication, we will hold it until you provide the relevant accession numbers or DOIs necessary to access your data. If you wish to make changes to your Data Availability statement, please describe these changes in your cover letter and we will update your Data Availability statement to reflect the information you provide.

[Reply] We will provide the relevant accession numbers or DOIs necessary to access our data.

3. PLOS requires an ORCID iD for the corresponding author in Editorial Manager on papers submitted after December 6th, 2016. Please ensure that you have an ORCID iD and that it is validated in Editorial Manager. To do this, go to ‘Update my Information’ (in the upper left-hand corner of the main menu), and click on the Fetch/Validate link next to the ORCID field. This will take you to the ORCID site and allow you to create a new iD or authenticate a pre-existing iD in Editorial Manager. Please see the following video for instructions on linking an ORCID iD to your Editorial Manager account: [https://www.youtube.com/watch?v=_xcclfuvtxQ](https://www.youtube.com/watch?v=_xcclfuvtxQ)

[Reply] We create a new iD:https://orcid.org/0000-0002-1699-7648

**COMMENTS FOR THE AUTHOR:**

**Reviewer 1#**

- The study is carefully concepted and methodological approach is satisfactory explained. The manuscript deals with interesting and important changes in response to salt stress on the photosynthetic physiology and mineral ion absorption and distribution in white willow (Salix alba L.). Results and discussion portions are well written. The authors also draw an accurate picture from the results.

[Reply] Thank you very much for your valuable feedback. We have made corrections based on the suggestions. Please see the manuscript.

- However, few parameters are missing. The authors should review and cite some more relevant references, and follow suggestion given in detail below.

[Reply] We have reviewed and cited more relevant references. Please see line 487-510 of the “Revised Manuscript with Track Changes”.

- Also needs a proper revision of English language. Many sentences are very confusing. English language and writing style needs to be improved sufficiently.

[Reply] We have modified the English language appropriately. Thank you very much for your valuable suggestions. We will continue our efforts to improve our English in the future.

- Do not repeat words of the title in the Keywords.

[Reply] We have rewrote Keywords. Please see line 41-42 of the “Revised Manuscript with Track Changes”.

- Please for the first use Salix alba L., then please follow the only Salix alba in the manuscript.

[Reply] We have rewrote the name throughout the manuscript. Please look at the example from line 15+18 of the “Revised Manuscript with Track Changes”.

- Add economic and other importance of plant in introduction to make it more valuable.

[Reply] We have added economic and other importance of plant in introduction. See Line 64-69 of the “Revised Manuscript with Track Changes” .

- It is better to NaCl concentration in mM instead of %.

[Reply] We've changed % to mM throughout the manuscript. Please see line 91 of the “Revised Manuscript with Track Changes”.

- Line: 25- 27. Please site the reference for hydroponics.

[Reply] We've cited the reference for hydroponics. Please see Line 89 +92-95+454-460 of the “Revised Manuscript with Track Changes” .

- Objective of the study needs to be refined.

[Reply] We have refined our research objectives. Please see Line 16-17 of the “Revised Manuscript with Track Changes”.

- Line: 98. Which instrument (its name model, company) used for the determination of ions?

[Reply] We have added information about this instrument. We used the atomic absorption spectrometer of Analytikjena in Germany for atomic absorption determination.Please see line 116-117 of the “Revised Manuscript with Track Changes”.

- In my opinion, chlorophyll content (a, b, and total) and carotenoids needs to be measured. It have direct link with salt stress and adaptability. NaCl and RWC have direct link when talking about stress and adaptability. So, I will suggest to study RWC (relative water content) under salt stress.

[Reply] This study mainly focuses on the effects of salt ions in plants on photosynthetic performance, without considering chlorophyⅡ and water. Thank you for your valuable suggestions, which will be added and improved in our future studies.

- References need to be revised. Many articles on salinity response are published in high impact factor journals. So try to cite them, so everyone can access the references as well. Also, try to cite the latest articles.

[Reply] We have deleted some references. We have added some influential and recent articles. Please see line 487-510 of the “Revised Manuscript with Track Changes”.

**COMMENTS FOR THE AUTHOR:**

**Reviewer 2#**

- However, this mechanism seems to be general in many plants, and also, as lack of data, the conclusions can not be rich and for further discussion.

[Reply] Thank you very much for your suggestions. We have revised the manuscript. Most of the researches on White Willow mainly focus on the medicinal value of substances such as salicin contained in the bark, or the value of studying the enrichment of heavy metals in white willow, which is mainly used to purify water resources and realize agricultural irrigation and fishery breeding. Habitat stress is mainly drought and flooding, but there are few literatures on salt stress, most of which focus on the responses of physiological indexes and photosynthetic indexes of plants to ion absorption and transport under salt stress. This is also one of the reasons for our research. We hope to use white willow as experimental material to observe the various effects of salt stress on plants from this perspective.

**COMMENTS FOR THE AUTHOR:**

**Reviewer 3#**

The manuscript titled “Effects of salt stress on the photosynthetic physiology and mineral ion absorption and distribution in white willow (Salix alba L.)” one of classic example of tree species response to salt stress. The manuscript is well organized and language easy to follow although there were few grammatical and Syntex errors besides SI units.

[Reply] Thank you very much for your suggestions. We have corrected grammatical and syntactic errors .Please see the manuscript.

-1. Adapting a hydroponic system to research salt stress may be the most recent best strategy, but there is no way of knowing how the experimental setting was done. Authors may include a photo of the same in order to make any relevant comments.

[Reply] We have added a picture to the manuscript. Please see the Figure 1.

-2.Similarly for rooting length, kindly provide some photo to understand plant response.

[Reply] We have added a photo. Please see the figure 2.

-3. Throughout the manuscript I can see that salt stress has a significant impact on above-ground biomass, such as leaf area index, shoot length, and so on, but the fact that it was not included in this study is a significant disadvantage. In the meantime, various physiological characteristics have been subjected leaves.

[Reply] This study mainly focuses on the effects of salt ions in plants on photosynthetic performance, without considering the aspects of leaves and stems. Thank you for your valuable suggestions, which will be added and improved in future research.

-4. When it comes to hydroponic systems, CK medium may be the best option, however, the reaction of Salix alba seedlings is substantially higher than 0.1% NaCl, but no justification from authors how or why although the salt concentration less than 0.1% NaCl.

[Reply] we have made an explanation, please see line 263-269 of the “Revised Manuscript with Track Changes”. Thank you very much for your valuable suggestions.

-5. A statistical analysis was performed, however it was not up to scientific merit. For example, the manuscript frequently mentions significance of attributes, but there are no ANOVA tables (except LSD rank), at least as a supplemental to support. I request the authors to include F=xxx, df=xxx, and sig.=>0.001 wherever statistical significance was indicated.

[Reply] We added some these information throughout the manuscript. Please see the example form line 173-175+179+181 of the “Revised Manuscript with Track Changes”.

6. Throughout the article, there are discussions about the trends of the morphological and physiological response of plant to the treatments, but the presented table does not provide viewpoints. Authors profusely mentioned the response trends widely without statistical analysis about trends which containing the scatter plot and best fit regression models (r2 and P). I advise authors to use graphs instead of tables, and I've included a sample presentation for ready reference.

[Reply] Thank you very much for your valuable suggestions. We have changed some tables into charts. Please see figure 7.
