# Supplementary material for: Effects of salt stress on the photosynthetic physiology and mineral ion absorption and distribution in white willow (Salix alba L.)
Source: PLoS One. 2021 Nov 18;16(11):e0260086. doi: 10.1371/journal.pone.0260086 (PMC8601552; doi:10.1371/journal.pone.0260086)
Supplement: Attachment — Submitted filename: Response to Reviewers.doc [file pone.0260086.s005.doc]

**Reply to all comments point-by-point (Manuscript Number: PONE-D-21-23173)**

The authors’ replies are in blue.

**Academic Editor’s comments:**

Dear Mayank Gururani Academic Editor,

Thank you very much for checking our submission on our manuscript(Manuscript Number: PONE-D-21-23173). Based on your comment and request, we have made extensive modification on the original manuscript.

1-Please include the following items when submitting your revised manuscript:

A rebuttal letter that responds to each point raised by the academic editor and reviewer(s). You should upload this letter as a separate file labeled 'Response to Reviewers'.A marked-up copy of your manuscript that highlights changes made to the original version. You should upload this as a separate file labeled 'Revised Manuscript with Track Changes'.An unmarked version of your revised paper without tracked changes. You should upload this as a separate file labeled 'Manuscript'.

[Reply]Thank you very much for your advice.We have submitted these three documents.

2-If you would like to make changes to your financial disclosure, please include your updated statement in your cover letter. Guidelines for resubmitting your figure files are available below the reviewer comments at the end of this letter.

[Reply]Thank you very much for your advice. We changed the pictures according to the guidelines.We also checked our images with PACE.

3-Please review your reference list to ensure that it is complete and correct. If you have cited papers that have been retracted, please include the rationale for doing so in the manuscript text, or remove these references and replace them with relevant current references. Any changes to the reference list should be mentioned in the rebuttal letter that accompanies your revised manuscript. If you need to cite a retracted article, indicate the article’s retracted status in the References list and also include a citation and full reference for the retraction notice.

[Reply]Thank you very much for your advice. We have checked our references.

Thank you very much for you giving us the opportunity to submit our revised manuscript. We look forward to hearing from you soon.

Best regards,

Sincerely yours,

Bingxiang Liu

**REVIEW COMMENTS TO THE AUTHOR:**

**Reviewer 1#**

1-All the comments have been addressed. Few minor corrections and suggestion are given below, which needs to be improve.

[Reply] Thank you very much for your valuable suggestions. We have corrected the question you raised. Please look at the following words.

2-Proper writing style is “Salix alba L.”, not “Salix alba. L”. Correct it and L. should not be italic (keywords and introduction). Also delete the dot after alba “Salix alba.”

[Reply] Thank you very much for your advice. We have corrected this writing error in its entirety. We changed ‘*Salix alba. L.* ’ to ‘ *Salix alba* L. ’ You can look at line 13 for an example.

We used *Salix alba* L. at the beginning of the manuscript and *Salix alba* later.

3-Line 40-41: you can cite the 10.3390/antiox9100940 manuscript for latest update about the Chinese areas affected with salinity. For global scenario cite 10.3389/fpls.2021.660409 manuscript.

[Reply] Thank you for providing us with these two articles, they are very helpful to our article, we have quoted them in line 42-44+421-424.

4-No need to write mM/L. mM is explaining the unit by itself.

[Reply] We have changed mM/L for mM throughout the paper.You can look at line 19 for an example.Thank you very much for your suggestions.

5-Still there are many typing and spelling mistakes. Revise the manuscript thoroughly.

[Reply] Thank you for your advice. We have revised the manuscript thoroughly. We will continue to study English hard in the future.

Finally special thanks to you for your valuable advice. Every suggestion you make will make our manuscript better.

**REVIEW COMMENTS TO THE AUTHOR:**

Reviewer 3#

1-The author has improved the revised manuscript in numerous ways, but it still lacks scientific merit, novelty, and grammatical errors, in addition to serious issues with SI units.Despite the authors' efforts, to revise the manuscript failed to address the serious concerns of reviewers regarding the experiment's leaf/above ground biomass to support the research.

[Reply] Thank you very much for your valuable suggestions.We have changed the error according to the SI units. Most of the researches on White Willow mainly focus on the medicinal value of substances such as salicin contained in the bark, or the value of studying the enrichment of heavy metals in white willow, which is mainly used to purify water resources and realize agricultural irrigation and fishery breeding. Habitat stress is mainly drought and flooding, but there are few literatures on salt stress, most of which focus on the responses of physiological indexes and photosynthetic indexes of plants to ion absorption and transport under salt stress. This is also one of the reasons for our research. We hope to use white willow as experimental material to observe the various effects of salt stress on plants from this perspective.

In this study, water culture was used to set different salinity gradient, and hard branch cutting was used to analyze the rooting of willow in different salinity. All these measures were to provide theoretical support for willow seedling cultivation in saline-alkali land. In this study, the growth of root system was the key factor to the survival of seedlings, so the rooting index was used in this study. The aboveground biomass can not be used to evaluate the condition of the seedlings because the shoots will die even if they do not take root.

2-Next is statistics and graphs part. The authors made a few changes, but the manuscript required extensive revision. They modified Fig 7, but similar revisions are required for all graphs except Figs 1, 2, and 10, which must be included in the manuscript.

[Reply] Thank you for your advice. We have modified Figs 3,4,5,6,8,9,11,12 and 13. We also used PACE to check our figures.

3-The anova results have been included in 165-175 lines, but they must be given wherever the word significant is used.

[Reply] Thank you very much for your valuable suggestions.We put these data next to the word ‘significant’. Please see line 177-186.

4-Figures are should be modified, XY axis legend and labels have to be edited carefully.

[Reply]Thank you very much for your suggestions. We revised all our Figures according to the journal's requirements.

5-The pdf file enclosed contains a list of all typos, grammatical errors, and suggestions.

[Reply]Thank you very much for your valuable advice. We corrected these errors according to this PDF file.

We will continue to improve our English in the future. Once again, thank you very much for your valuable suggestions..

We appreciate for Editors and reviewers’ warm work earnestly, and hope that the corrections will meet with approval.
